# Human Adenovirus-Specific γ/δ and CD8^+^ T Cells Generated by T-Cell Receptor Transfection to Treat Adenovirus Infection after Allogeneic Stem Cell Transplantation

**DOI:** 10.1371/journal.pone.0109944

**Published:** 2014-10-07

**Authors:** Jan Dörrie, Christian Krug, Christian Hofmann, Ina Müller, Verena Wellner, Ilka Knippertz, Stephan Schierer, Simone Thomas, Elke Zipperer, Dieter Printz, Gerhard Fritsch, Gerold Schuler, Niels Schaft, Rene Geyeregger

**Affiliations:** 1 Department of Dermatology, Universitätsklinikum Erlangen, Erlangen, Germany; 2 Department of Immune Modulation at the Dept. of Dermatology, Universitätsklinikum Erlangen, Erlangen, Germany; 3 St. Anna Kinderkrebsforschung e.V., Children's Cancer Research Institute, Vienna, Austria; 4 Department of Pediatrics, Medical University of Vienna, Vienna, Austria; 5 Department of Internal Medicine III, University Hospital of Regensburg, Regensburg, Germany; 6 Friedrich-Alexander-Universität Erlangen-Nürnberg, Erlangen, Germany; Leiden University Medical Center, Netherlands

## Abstract

Human adenovirus infection is life threatening after allogeneic haematopoietic stem cell transplantation (HSCT). Immunotherapy with donor-derived adenovirus-specific T cells is promising; however, 20% of all donors lack adenovirus-specific T cells. To overcome this, we transfected α/β T cells with mRNA encoding a T-cell receptor (TCR) specific for the HLA-A*0101-restricted peptide LTDLGQNLLY from the adenovirus hexon protein. Furthermore, since allo-reactive endogenous TCR of donor T lymphocytes would induce graft-versus-host disease (GvHD) in a mismatched patient, we transferred the TCR into γ/δ T cells, which are not allo-reactive. TCR-transfected γ/δ T cells secreted low quantities of cytokines after antigen-specific stimulation, which were increased dramatically after co-transfection of CD8α-encoding mRNA. In direct comparison with TCR-transfected α/β T cells, TCR-CD8α-co-transfected γ/δ T cells produced more tumor necrosis factor (TNF), and lysed peptide-loaded target cells as efficiently. Most importantly, TCR-transfected α/β T cells and TCR-CD8α-co-transfected γ/δ T cells efficiently lysed adenovirus-infected target cells. We show here, for the first time, that not only α/β T cells but also γ/δ T cells can be equipped with an adenovirus specificity by TCR-RNA electroporation. Thus, our strategy offers a new means for the immunotherapy of adenovirus infection after allogeneic HSCT.

## Introduction

After allogeneic haematopoietic stem cell transplantation (HSCT) human adenovirus (HAdV) infection is a life threatening complication. The overall HAdV-associated mortality ranges from 18 to 26% [1] and mortality rates of 14 to 100% in infected patients despite virostatic treatment are described [2]. Additionally, treatment with antiviral drugs is associated with substantial nephron- and myelotoxicity [3].

Immunotherapy with either magnetically separated [4] or expanded [5] HAdV-specific T cells represents a promising treatment option to overcome viral infections after allogeneic HSCT. More recent approaches are based on the short-term expansion of HAdV-specific T cells with overlapping 15-mer polypeptides from highly conserved regions of the immunodominant major capsid protein hexon [6], [7], to facilitate broad recognition and protection against several HAdV species [8].

However, as a prerequisite for such immunotherapies, the T-cell donor has to have virus-specific T cells. Recent data from our laboratory showed that in 12 out of 50 donors, no HAdV-specific T cells were detectable via MHC class I multimers and/or IFNγ ELIspot (unpublished data). Although the serotype was not analysed, this is in accordance with the generally high prevalence (<80%) of the common species C HAdV infection in the human population [9], with some geographic variations between 40% of adults in America [10], 93% of children in Sub-Saharan Africa [11], and about 77% in southern China [12]. Due to the incomplete match of donor and recipient, the use of donor T cells is further restricted because they only react in the presence of matching HLA molecules.

One alternative would be the transfer of T-cell receptors (TCR) with defined antigen specificities to peripheral blood T cells [13]. TCR specific for tumor antigens were already effectively transferred in several animal models [14]–[16] and at least in one clinical phase I/II study [17]. To treat CMV-infections, the use of TCR-redirected CMV-specific T cells was recently discussed [18]. Although several CMV-specific TCR are already known, no HAdV-specific TCR have been identified until now.

In contrast to retroviral transduction, mRNA electroporation avoids potential severe side effects by inducing only transient expression of the exogenous TCR, lasting several days [19]. However, this implies multiple infusions of high cell numbers. Recently, it was shown that despite transient functionality, the TCR electroporated T cells were able to efficiently prevent tumor seeding and suppress tumor growth in a xenograft model of hepatocellular carcinoma [20]. Because the period during which an HSCT recipient suffers complete immunosuppression is temporary, we consider this setting well suitable for the use of mRNA-transfected T cells.

The infusion of donor-derived TCR-redirected α/β T cells would, therefore, be a possible treatment strategy for HLA-matched patients suffering of severe HAdV complications [21]. Nevertheless, the number of donor-derived α/β T cells that can be infused into HLA-mismatched patients post HSCT is limited, as these cells exhibit allo-reactivity via their endogenous TCR.

This could be overcome by using γ/δ T cells, which do not recognize MHC molecules and are hence not allo-reactive [22]. It was shown that γ/δ T cells – retrovirally transfected with α/β TCR against e.g. CMV or a tumor antigen- were highly functional in vitro [23] and in mice [24], [25].

In this study we expanded HAdV-specific T cells by stimulation with the HLA-A*0101-restricted, immunodominant, and cross-reactive epitope LTDLGQNLLY (LTD) from the hexon protein of HAdV-species C, the predominant species in patients after HSCT [1]. We identified, for the first time, HAdV-specific TCR α/β chain sequences, which were then cloned and transfected via mRNA electroporation into CD8^+^ α/β T cells and γ/δ T cells and tested for their capability to induce cytokine secretion and lysis of peptide-loaded, or adenovirus-infected target cells. Therefore, we report here on a new therapeutic possibility for the treatment of HAdV infection after allogeneic stem cell transplantation with HAdV-TCR-transfected CD8^+^ α/β T cells and γ/δ T cells.

## Materials and Methods

### Cells and reagents

All human material was obtained following written informed consent and approved by the institutional review board in Erlangen (Ethik-Kommission der Medizinischen Fakultät der Friedrich-Alexander-Universtität Erlangen-Nürnberg, #3928) and the Ethic Committee in Vienna (Ethik-Kommission der Medizinischen Universität Wien, EK Nr. 514/2011), and all investigations were conducted according to the principles expressed in the Declaration of Helsinki. PBMC of healthy volunteers were prepared by density centrifugation using Lymphoprep (Axis-Shield, Oslo, Norway). CD8^+^ α/β T cells were isolated from PBMC using anti-CD8 MACS beads according to the manufacturer's instructions (Miltenyi Biotec), and were cultured in MLPC medium consisting of RPMI 1640 (Lonza), 10% human serum (Lonza), 2 mM L-glutamine (Lonza), 20 mg/L gentamicin (Sigma-Aldrich), 10 mM HEPES (PAA, GE healthcare), 1 mM sodium pyruvate (Sigma-Aldrich), and 1% MEM nonessential amino acids (100x; PAA, GE healthcare), supplemented with 1000 IU/ml IL-2 (Proleukin; Novartis) and 10 ng/ml IL-7 (Peprotech). In some experiments expanded CD8^+^ α/β T cells and γ/δ T cells were used basically as described previously [26]. In short, PBMC were cultured at 1×10^6^ cells/ml in R10 medium consisting of RPMI 1640 containing final concentrations of 10% (v/v) heat-inactivated fetal bovine serum (PAA, GE healthcare), 2 mM L-glutamine, 100 U/ml penicillin, 100 µg/ml streptomycin (Lonza), 2 mM HEPES, and 20 µM β-mercaptoethanol (Gibco, Life Technologies). A final concentration of 0.1 µg/ml anti-CD3 antibody (Orthoclone OKT-3; Jannsen-Cilag) and 10^3^ IU/ml IL-2 were added on day 0. Fresh IL-2 (10^3^ IU/ml) was added on day 2. On day 3, the cells were counted and diluted to 0.2×10^6^ cells/ml in R10 medium and 10^3^ IU/ml IL-2 were added. The same amount of IL-2 was added on day 5 of culture. The total culture volume was doubled by adding fresh medium on day 7, and IL-2 (10^3^ IU/ml) was added as well. Cells were harvested after nine days of expansion and subsequently CD8^+^ α/β T cells and γ/δ T cells were isolated from PBMC using anti-CD8 MACS beads and the TCRγ/δ^+^ T Cell Isolation Kit, respectively, according to the manufacturer's instructions (Miltenyi Biotec). The cells were cultured in R10 medium overnight. Mature DC were generated as described [27]. The melanoma cell line colo829 (acquired from ATCC) (HLA-A1^+^) was cultured in R10 medium. The EBV-transformed cell line CCL (HLA-A1^+^; published before by Felzmann et al. [28]) was cultured in R20, which is R10 medium containing 20% (v/v) heat-inactivated fetal bovine serum instead of 10%. HLA-A1-binding peptides used in this study were HAdV: LTDLGQNLLY, MAGE-A3: EVDPIGHLY, and MAGE-A1: EADPTGHSY (Eurogentec).

#### Isolation of ADV-specific oligoclonal T cells

In total, 160×10^6^ PBMC (10^7^/ml) from an HLA-type A*0101-positive donor were cultured in AIM-V (Invitrogen, Carlsbad, CA) supplemented with 2% Octaplas (Octapharma, Vienna, Austria), 2 mM L-Glutamine, and 25 mM HEPES, and were stimulated for 6 days with HAdV (subgroup C-derived Hexon AdV5)-specific peptide pools (Miltenyi Biotec) at a final concentration of 0.6 nmol for each peptide per ml. On day 6, cultured cells were added to adherent monocytes (as described in [6]) and re-stimulated with the peptide pool and IL-2 (R&D Systems) at 5 ng/ml. On day 12, 10^8^ cells were washed, stained with 500 µl PE-labeled A*0101-ADV-specific pentamers (Proimmune), incubated with anti-PE MicroBeads (Miltenyi Biotec), and isolated according to the manufacturer's instructions. As a next step, highly pure PE-pentamer^+^ T cells were sorted by flow cytometry and resuspended in TRIzol (Invitrogen).

### Cloning of TCR genes and in vitro transcription of mRNA

The subtypes of TCR α and β chain of the HLA-A1/adenovirus-specific CTL clone, which was most prominent in the isolated oligoclonal T cells were identified as described previously [29]. The TCR α chain was of the AV30S1AT [30]/AV20 (IMGT) subtype, and the TCR β chain was of the BV4SA1T [30]/BV29-1 (IMGT) subtype. The full length TCR chains were cloned into a pGEM4Z-5′UTR-sig-huSurvivin-DC.LAMP-3′UTR vector, replacing the huSurvivin-DC.LAMP. As controls, an HLA-A1/MAGE-A3-specific TCR and an HLA-A1/MAGE-A1-specific TCR were used. In vitro transcriptions of TCR RNA were performed using mMESSAGE mMACHINE T7 ULTRA kits (Life technologies) according to the manufacturer's instructions.

#### Jurkat T cell/luciferase assay

The Jurkat T cell (acquired from ATCC)/luciferase assay was performed as described previously [29]. As target cells DC, which were loaded for 1 h at 37°C with the indicated peptides (all at 10 µg/ml), or were left unloaded, were used. To determine antigen-specific luciferase production, the luciferase activity was set in relation to the luciferase activity measured with the non-loaded target cells as stimulators.

### RNA electroporation of T lymphocytes

CD8^+^ α/β T cells and γ/δ T cells were electroporated with the following settings: square-wave pulse, 500 V, 3 ms or 5 ms, as described previously [31].

### Adenoviruses

The replication-deficient Ad5Luc1 virus was amplified in 293 T cells (acquired from ATCC) as described before [32]. Non-replication restricted Ad5wt was amplified in colo829 cells [33].

#### Cell surface marker and TCR staining

Thawed T cells transfected with either the control TCR specific for MAGE1/A1 or the HAdV/A1 TCR were washed in FACS-buffer and 0.25×10^6^ cells per condition were stained using the following antibodies: IgG1-FITC (BD Biosciences), IgG2a-FITC (BD Biosciences), anti-γ/δ pan TCR-FITC (Pierce Antibody products, Thermo Scientific), anti-CD4-FITC (BD Biosciences), anti-CD8-FITC (BD Biosciences), anti-CD14-FITC (BD Biosciences), anti-CD16-FITC (BD Biosciences), anti-CD19-FITC (BD Biosciences) in 50 µl of FACS-buffer for 30 min at 4°C. Cells were then washed once and analyzed on a FACScan (BD Biosciences). For staining of HAdV-TCR-transfected T lymphocytes via MHC I streptamers, in total 0.25×10^6^ T cells were washed and then resuspended in 50 µl buffer. Cells were then incubated with streptamers comprising the HLA-A1 (LTDLGQNLLY) MHC class I (1 µl) and Strep-Tactin-PE (1.25 µl) (IBA GmBH, Göttingen, Germany) for 30 min at 4°C according to manufacturer's instructions. After washing, cells were resuspended in buffer and analyzed by flow-cytometry.

#### Induction and determination of cytokine production by TCR-transfected T lymphocytes

T cells electroporated with TCR-encoding mRNA were co-cultivated with the melanoma cell line colo829, EBV-transformed B cells (CCL), or DC (all HLA-A1^+^) that were either non-loaded or loaded with a control peptide or the peptide recognized by the ADV-specific TCR (LTDLGQNLLY) (all at 10 µg/ml) for 1 h at 37°C. Alternatively, HAdV-infected target cells were used. Therefore, 4×10^6^ DC were seeded in 6-well plates (Falcon) followed by transduction with adenovirus at 5000–10000 viral particles/ml (vp/ml) in a final volume of 1 ml R10 medium containing additionally 550 U/ml GM-CSF and 800 U/ml IL-4 (both Miltenyi Biotec). After 1.5 hours of incubation at room temperature on a rocker, 4 ml of growth medium replenished with cytokines as described before was added per well. Cells were incubated for 48 h at 37°C, 5% CO_2_, before they were used for further experiments. Transduction efficacy (GFP expression) and percentage of living cells was determined by flow cytometric analysis with a FACScan cell analyzer (BD Biosciences). Cytokine production was determined as described previously [34]. Alternatively, intracellular cytokine staining was performed as described before [35]. In short, a total of 1×10^6^ T cells were stimulated with 1×10^6^ non-peptide-loaded or HAdV-peptide-loaded CCL cells in 500 µl MLPC with 2.5 µg BrefeldinA (Sigma-Aldrich) and 373 ng monensin (Sigma-Aldrich) for approximately 12 h. Cells were stained with Live/Dead aqua-mix (Invitrogen) and were extracellularly stained with αCD8-PerCP- (BD Biosciences), αCD4-V450- (BD Biosciences), and αCD14-Pacific Orange antibodies (Invitrogen). Subsequently, cells were fixed and permeabilized with reagents from eBioscience, and intracellularly stained with αCD8-PerCP- (BD Biosciences), αIL-2-APC- (BD Biosciences), αTNF-PE-Cy7- (BD Biosciences), and IFNγ-Alexa Fluor 700 (BD Biosciences) antibodies. Cells were measured with the FACS Canto II (BD Biosciences) and analyzed with FCS Express 4 Software (De Novo Software).

### Cytotoxicity assay

Cytotoxicity was tested in standard 4–6 h ^51^Cr release assays as described previously [31]. Peptide-loaded and HAdV-infected target cells were used. Percentage cytolysis, i.e., ^51^Cr release, was calculated as follows: [(measured release – background release)]/[(maximum release with 1% triton – background release)] ×100%.

### Statistics

Statistical analysis was performed using the Graph Pad Prism software. P-values were calculated by the Mann-Whitney U test.

## Results

### The newly cloned HAdV/HLA-A1-specific TCR can be functionally transferred to T cells

**Figure 1:**
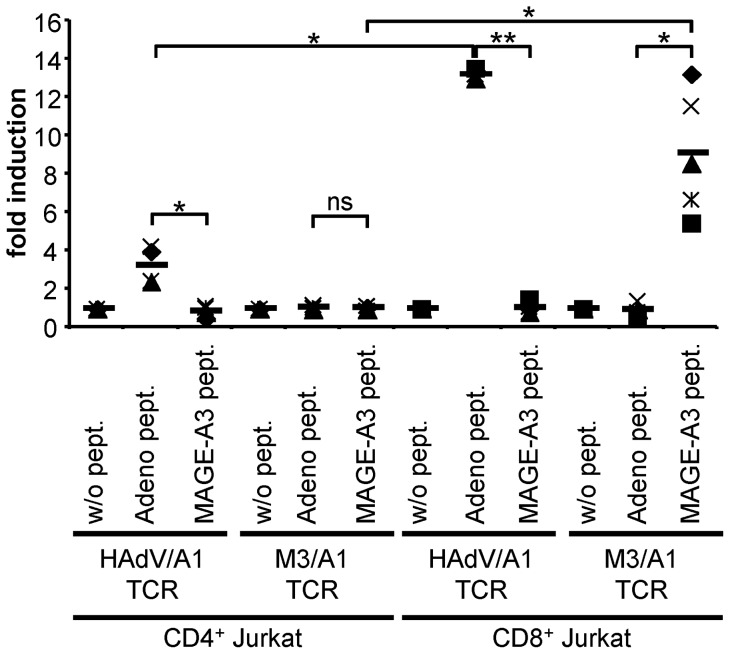
The newly cloned HAdV/HLA-A1-specific TCR is functional in Jurkat T cells. Parental CD4^+^ and transgenic CD8^+^ Jurkat T cells were co-electroporated with RNA encoding the HAdV/A1-specific TCR or MAGE-A3/A1 (M3/A1)-specific TCR and an NFAT-inducible luciferase reporter plasmid. These Jurkat T cells were stimulated with DC either non-loaded (w/o pept.) or loaded with the adenovirus peptide (Adeno pept.) or the MAGE-A3 peptide (MAGE-A3 pept.) (as indicated). The luciferase activity was measured, and the specific activation of the Jurkat T cells was calculated as fold induction by dividing the luciferase activity induced by peptide-loaded DC by that of similarly electroporated Jurkat T cells stimulated with non-loaded DC. Data of 4 (CD4^+^ Jurkat T cells) and 5 (CD8^+^ Jurkat T cells) individual experiments are shown. Bars indicate mean values. P-values were calculated by the Mann-Whitney U test. ns  =  not significant; ** p≤0.01; * p≤0.05. Raw data are summarized in Table S1 in File S1.

To investigate the functional transfer of a HAdV/HLA-A1 (HAdV/A1)-specific TCR, we first cloned such a TCR from a CD8^+^ CTL clone, and tested it in a Jurkat T cell/luciferase assay [29]. Jurkat cells were co-electroporated with RNA encoding the TCR and the DNA Transluc vector that encodes luciferase under the control of an NFAT-inducible promoter. As a control mRNA encoding a MAGE-A3/HLA-A1 (M3/A1)-specific TCR was used in parallel. These cells were co-cultured with HLA-A1-positive dendritic cells (DC) loaded with either the adenovirus peptide or the MAGE-A3 peptide. As a negative control, DC were left unloaded. Then luciferase activity induced by an antigen-specific TCR signal was determined (Fig. 1). To examine the TCR's dependence on the co-receptor CD8, a CD8-transgenic Jurkat derivative was used in addition. In these CD8^+^ Jurkat T cells, the transferred HAdV/A1-specific TCR did not recognize the non-loaded DC or MAGE-A3-peptide-loaded DC, while it recognized the adenovirus peptide specifically (Fig. 1). CD8^+^ Jurkat cells transfected with the M3/A1-specific control TCR only recognized the MAGE-A3-peptide-loaded DC (Fig. 1). Even in the CD8-negative parental Jurkat T cells the HAdV/A1-specific TCR recognized adenovirus-peptide-loaded DC specifically, while the M3/A1-specific TCR did not (Fig. 1).

Taken together, these data indicate that the cloned TCR is functional and HAdV/A1-specific, and binds its target in a partially CD8 independent manner.

### HAdV/A1-TCR-transfected CD8^+^ T cells recognize target cells antigen-specifically

**Figure 2:**
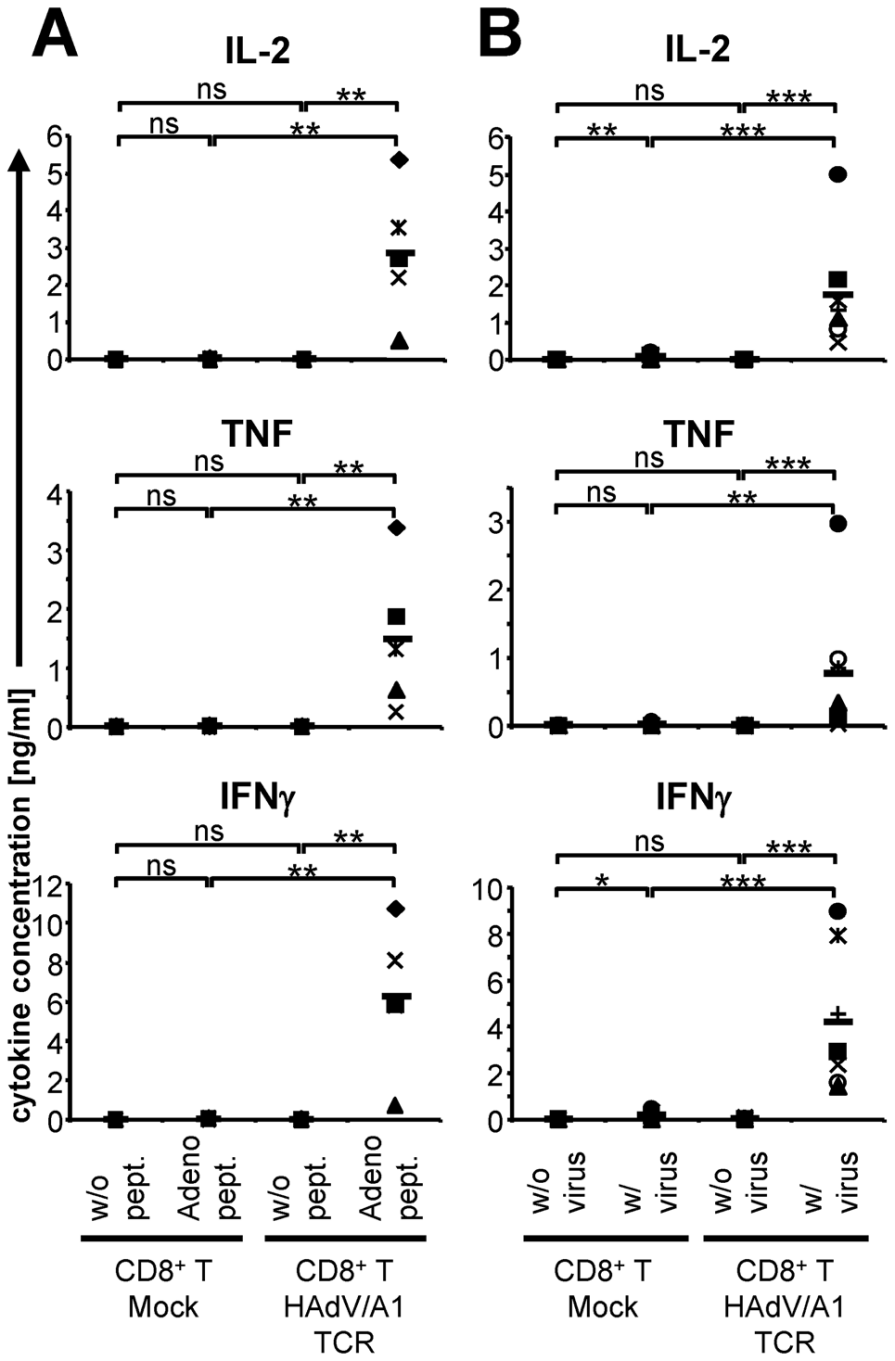
Antigen-specific cytokine production by HAdV/A1-TCR-transfected CD8^+^ T cells in response to peptide-loaded and adenovirus-infected targets. CD8^+^ T cells were either mock electroporated or electroporated with HAdV/A1-TCR-RNA and were stimulated with DC, which were either left unloaded (**A**: w/o pept., **B**: w/o virus), or were loaded with the adenovirus peptide (Adeno pept.) (**A**), or were infected with adenovirus (w/virus) (**B**). Cytokine concentrations (IL-2, TNF, and IFNγ) in the supernatant after over-night co-incubation are depicted. Data of 5 (**A**) and 7 (**B**) individual experiments are shown. Bars indicate mean values. P-values were calculated by the Mann-Whitney U test. ns  =  not significant, *** p≤0.001; ** p≤0.01; * p≤0.05. Raw data are summarized in Table S2A and B in File S1.

To study the functionality of the cloned HAdV/A1-specific TCR in primary T cells, we transferred the TCR to CD8^+^ T cells isolated from healthy donor blood by mRNA electroporation. The TCR-transfected CD8^+^ T cells were incubated with peptide-loaded HLA-A1^+^ DC overnight, and cytokine production was determined. Only HAdV/A1-TCR-transfected CD8^+^ T cells recognized the adenovirus-peptide-loaded DC and responded with IL-2, TNF, and IFNγ production (Fig. 2A). Non-loaded DC were not recognized by these T cells, and mock-electroporated CD8^+^ T cells did not produce cytokines after incubation with non-loaded or adenovirus-peptide-loaded DC (Fig. 2A).

Furthermore, the response of HAdV/A1-specific TCR-transfected CD8^+^ T cells to adenovirus-infected DC was investigated (Fig. 2B). Again, only the HAdV/A1-TCR-transfected CD8^+^ T cells recognized the adenovirus-infected DC and produced substantial amounts of IL-2, TNF, and IFNγ (Fig. 2B). Non-infected DC were not recognized, and mock-electroporated CD8^+^ T cells did not produce any cytokines after incubation with non-infected DC. Marginal, but significant amounts of IL-2 and IFNγ were produced by mock-electroporated T cells in response to adenovirus-infected DC, probably due to a pre-existing adenoviral activity of the donor blood (Fig. 2B).

Taken together, CD8^+^ T cells transfected with the HAdV/A1-specific TCR by mRNA electroporation antigen-specifically secreted pro-inflammatory cytokines.

### HAdV/A1-TCR and CD8α co-transfected γ/δ T cells produce cytokines antigen-specifically

**Figure 3:**
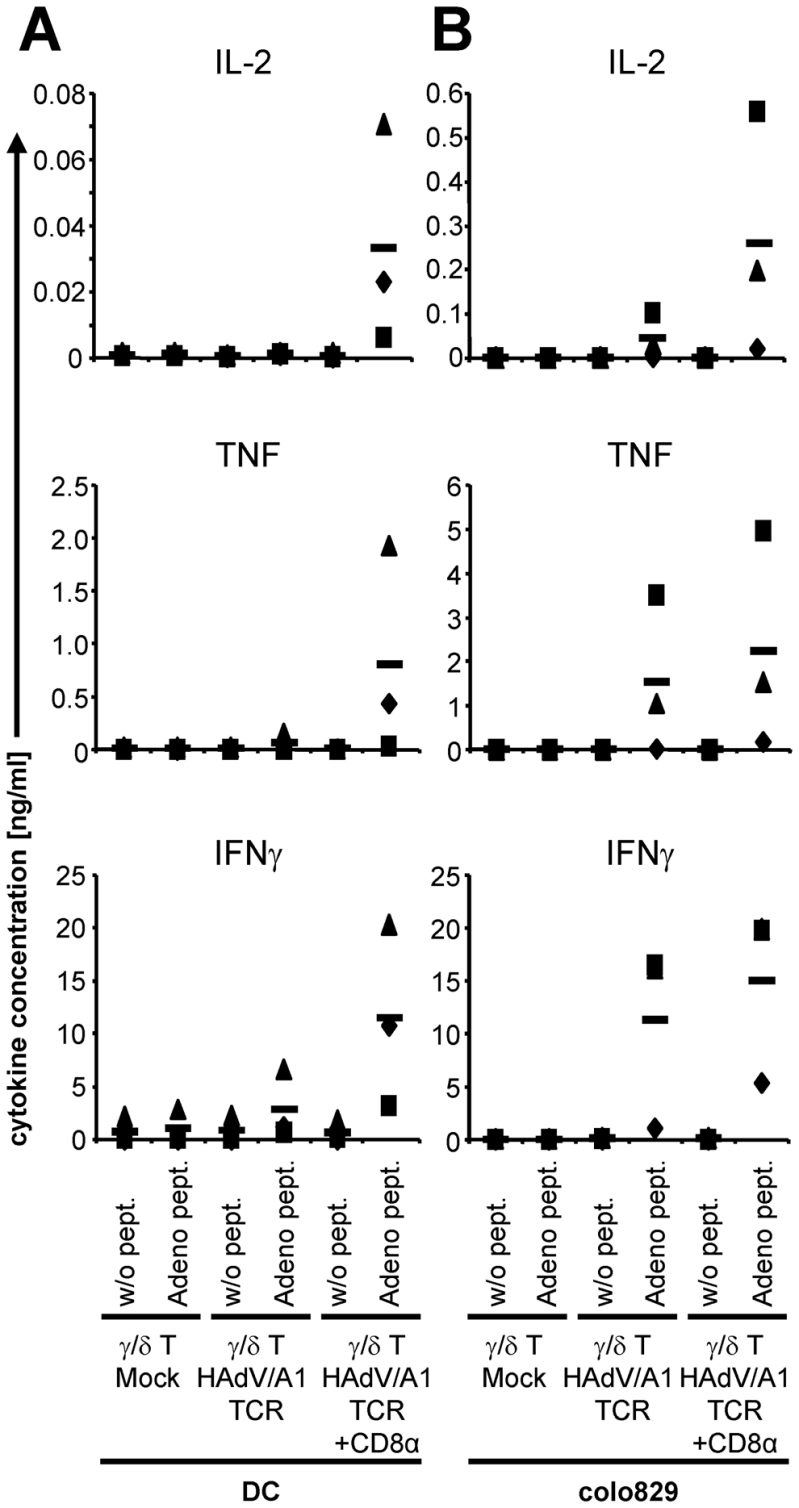
Antigen-specific cytokine production by HAdV/A1-TCR-transfected γ/δ T cells in response to peptide-loaded target cells. γ/δ T cells were either mock electroporated, electroporated with HAdV/A1-TCR-RNA alone, or electroporated with HAdV/A1-TCR-RNA and CD8α-RNA. These cells were stimulated with DC (**A**) or colo829 cells (**B**), which were either left unloaded (w/o pept.), or were loaded with the adenovirus peptide (Adeno pept.). Cytokine concentrations (IL-2, TNF, and IFNγ) in the supernatant after overnight co-incubation are depicted. Data of 3 individual experiments are shown. Bars indicate mean values. Raw data are summarized in Table S3A and B in in File S1.

Since the endogenous TCR of the donor T lymphocytes would induce GvHD in a mismatched patient, we investigated whether the HAdV/A1-specific TCR can be functionally transferred into not allo-reactive γ/δ T cells by mRNA electroporation. γ/δ T cells were isolated from expanded PBMC of healthy donors to obtain sufficient numbers for electroporation. TCR-transfected cells were incubated with peptide-loaded DC (Fig. 3A) or colo829, a melanoma cell line (Fig. 3B). The TCR-transfected γ/δ T cells produced minor amounts of IFNγ after stimulation with adenovirus-peptide-loaded DC (Fig. 3A). The response to adenovirus-peptide-loaded colo839 cells by TCR-transfected γ/δ T cells was higher; i.e. an antigen-specific secretion of IL-2, TNF, and IFNγ was clearly detected (Fig. 3B). As the HAdV/A1-TCR was cloned from a CD8^+^ T-cell clone, we reasoned that the TCR might require CD8 co-binding for optimal functionality. Hence, we co-transfected γ/δ T cells with the HAdV/A1-TCR and CD8α by mRNA electroporation and tested these cells on the same target cells as described above. This resulted in a clear increase in the antigen-specific cytokine secretion in response to adenovirus-peptide-loaded DC (Fig. 3A) and colo829 cells (Fig. 3B). TCR-transfected γ/δ T cells did not recognize non-loaded target cells, and mock-electroporated γ/δ T cells did not produce any cytokine (Fig. 3).

In summary, we were able to functionally transfer the HAdV/A1-TCR to γ/δ T cells by mRNA electroporation leading to an antigen-specific cytokine secretion. Co-transfection of TCR and CD8α into γ/δ T cells further increased the functionality of these cells.

### TCR-transfected γ/δ T cells appear to have a better functionality than TCR-transfected CD8^+^ T cells in direct comparison

**Figure 4:**
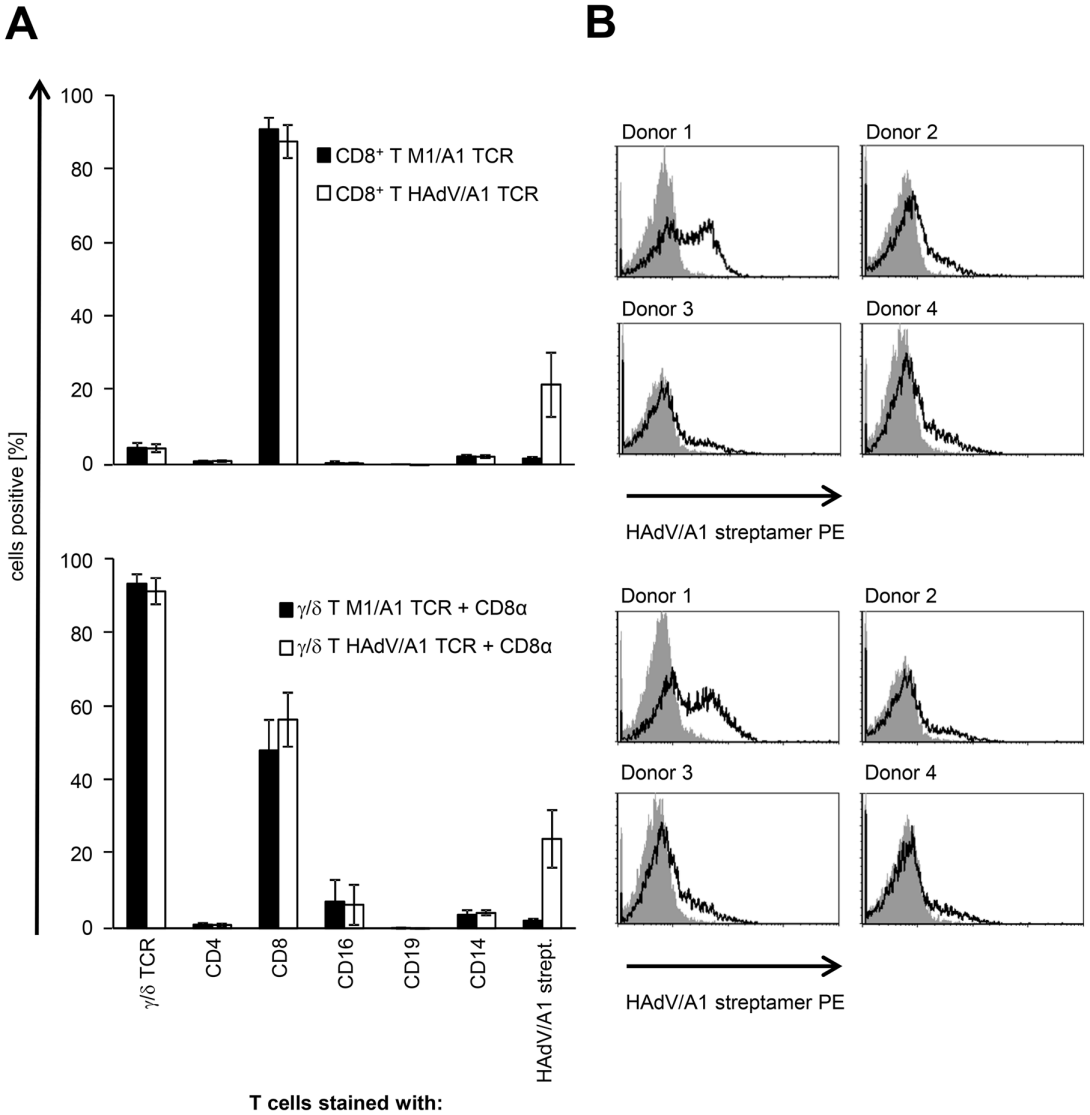
Surface marker and TCR expression on HAdV/A1-TCR-transfected CD8^+^ and γ/δ T cells. CD8^+^ T cells (A and B; upper panels) and γ/δ T cells (A and B; lower panels) were either electroporated with M1/A1-TCR RNA (+CD8α RNA in case of γ/δ T cells) or with HAdV/A1-TCR-RNA (+CD8α RNA in case of γ/δ T cells) and were cryoconserved 4 h after electroporation. Surface marker and TCR expression on thawed T cells was determined by staining with anti-CD4, anti-CD8, anti-CD16, anti-CD19, anti-CD14, and anti-pan γ/δ TCR antibodies, and the expression of the HAdV/A1-specific TCR was determined by staining with HAdV/A1 streptamer. Expression levels were analyzed by flow-cytometry and shown as % positive cells; isotype control stainings were subtracted (**A**; average values of 4 experiments ± SEM) or as histograms (**B**; grey histogram: T cells electroporated with M1/A1-TCR RNA, black line: T cells electroporated with HAdV/A1-TCR-RNA). Raw data are summarized in Table S4A and B in File S1.

To determine the reactivity of the TCR-transfected γ/δ T cells in comparison to TCR-transfected CD8^+^ T cells of the same donor, we directly compared these cells. Both T-cell populations were expanded and transfected with the HAdV/A1-TCR, and γ/δ T cells were co-transfected with CD8α. To check for purity of the isolated and expanded cells, expression of CD4, CD8, CD14, CD16, CD19, and γ/δ TCR on the cell surface were determined (Fig. 4A). Of the isolated and expanded CD8^+^ T cells, approximately 90% were CD8 positive, and only 4.5% were γ/δ TCR positive (Fig. 4A). Of the isolated and expanded γ/δ T cells, which were co-transfected with TCR and CD8α,>90% were γ/δ TCR positive, and approximately 50% were CD8 positive (Fig. 4A). To determine TCR-transfection efficiency, the RNA-transfected cells were stained with the HAdV/A1-streptamer (Fig. 4). In average, approximately 21% of the CD8^+^ T cells and 24% of the γ/δ T cells expressed the TCRα and TCRβ chain of the HAdV/A1-specific TCR efficiently enough to facilitate streptamer-binding (Fig. 4).

**Figure 5:**
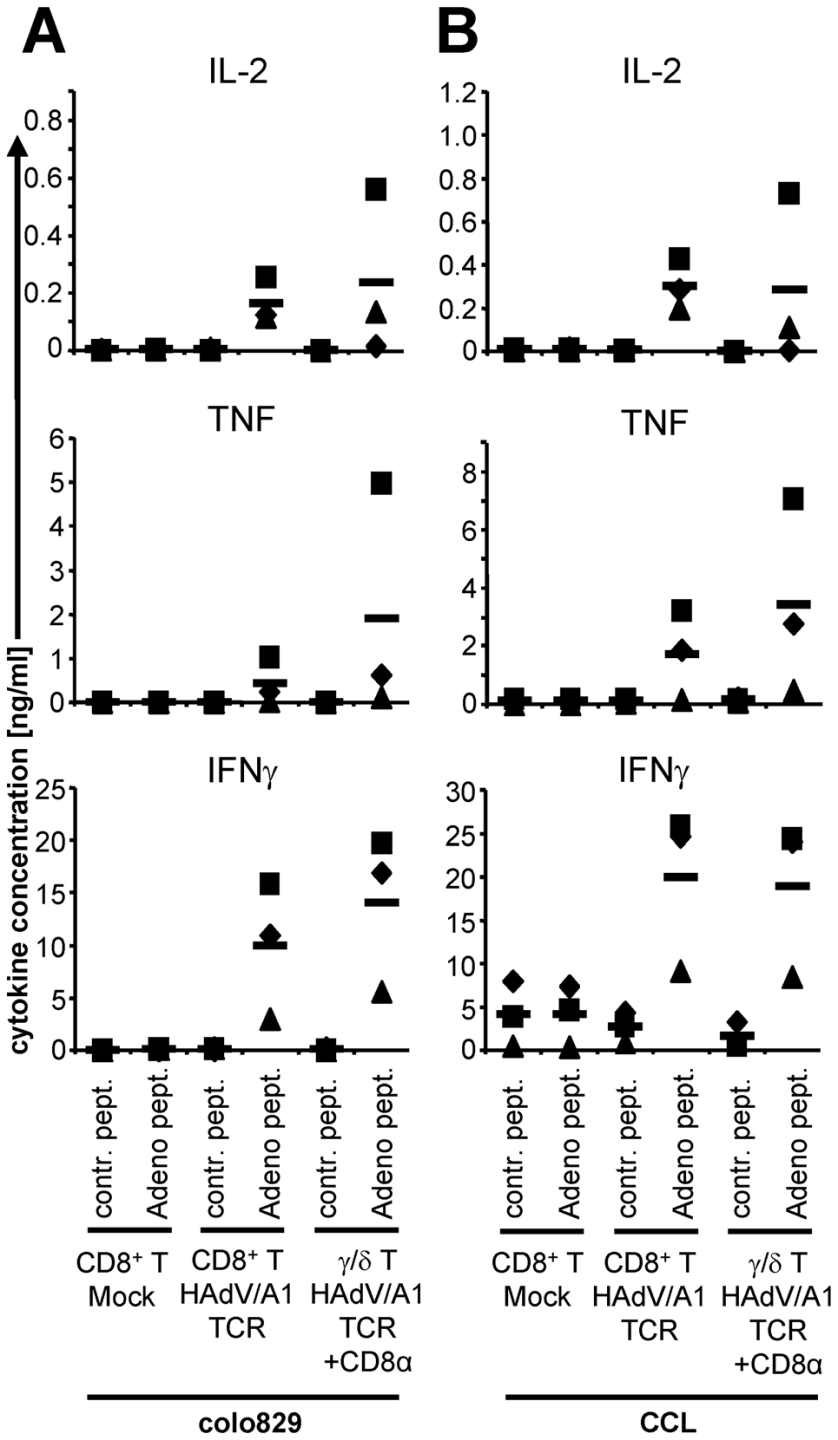
Direct comparison of cytokine production by HAdV/A1-TCR-transfected CD8^+^ T cells and HAdV/A1-TCR-transfected γ/δ T cells. CD8^+^ T cells and γ/δ T cells of the same donors were either mock electroporated, electroporated with HAdV/A1-TCR-RNA alone, or electroporated with HAdV/A1-TCR-RNA and CD8α-RNA. These cells were stimulated with colo829 cells (**A**) or CCL cells (**B**), which were either loaded with a control peptide (contr. pept.) or the adenovirus peptide (Adeno pept.). Cytokine concentrations (IL-2, TNF, and IFNγ) in the supernatant after overnight co-incubation are depicted. Data of 3 individual experiments are shown. Bars indicate mean values. Raw data are summarized in Table S5A and B in File S1.

**Figure 6:**
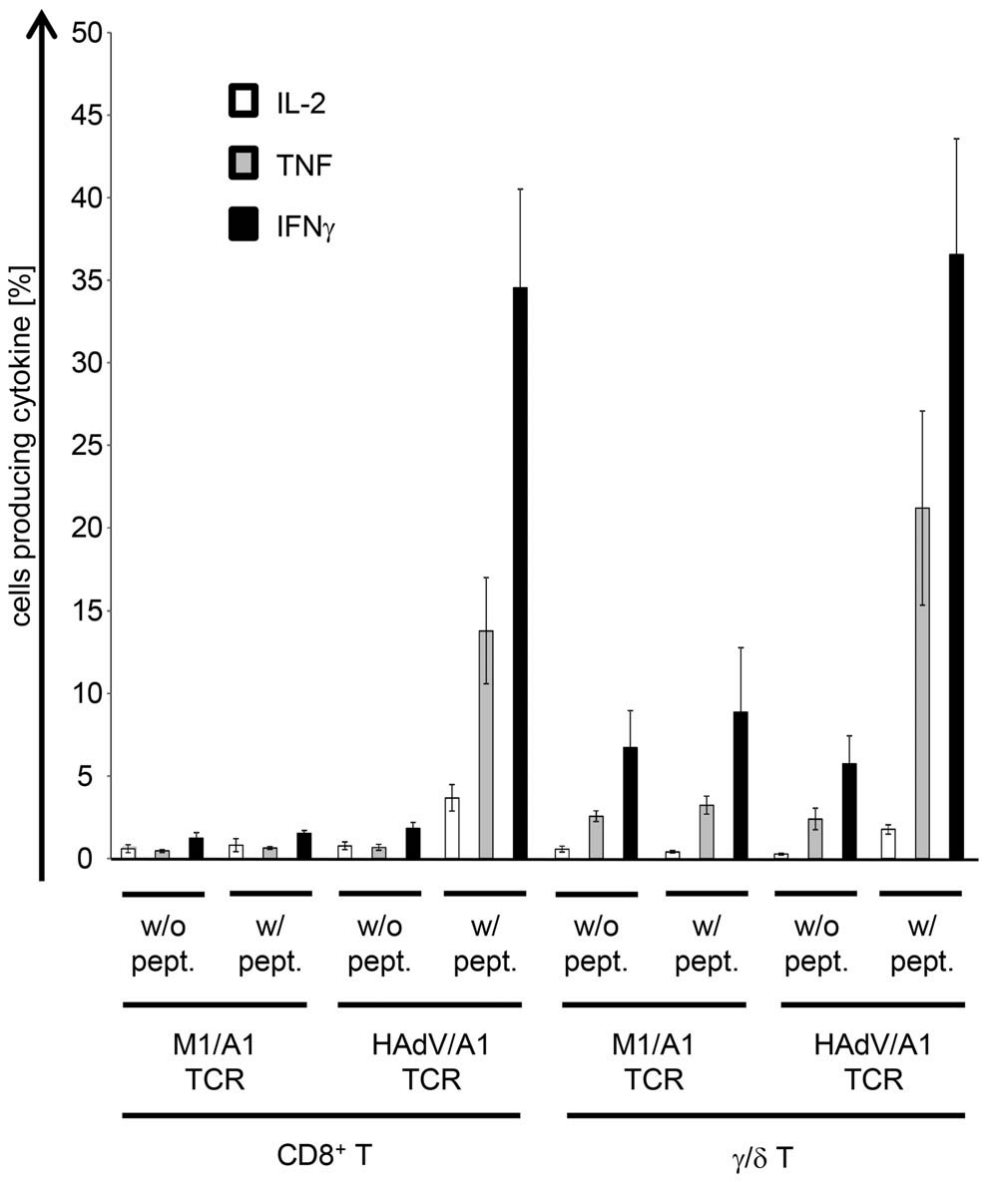
Antigen-specific cytokine production by HAdV/A1-TCR-transfected CD8^+^ and γ/δ T cells in response to peptide-loaded target cells. CD8^+^ T cells and γ/δ T cells were either electroporated with M1/A1-TCR RNA (+CD8α RNA in case of γ/δ T cells) or with HAdV/A1-TCR-RNA (+CD8α RNA in case of γ/δ T cells) and were cryoconserved 4 h after electroporation. After thawing, these cells were stimulated with CCL cells, which were either left unloaded (w/o pept.), or were loaded with the adenovirus peptide (w/pept.). Intracellular cytokine stainings for IL-2 (white bars), TNF (grey bars), and IFNγ (black bars) were performed and analyzed by flow-cytometry. The percentages of cytokine containing cells are depicted. Average values of 4 (CD8^+^ T cells) and 3 (γ/δ T cells) individual experiments ± SEM are shown. Raw data are summarized in Table S6 in File S1.

Then the TCR-transfected cells were incubated with peptide-loaded colo829 (Fig. 5A) or CCL cells (Fig. 5B and 6). Mock-electroporated CD8^+^ T cells did not produce any cytokines, or only at background levels (Fig. 5). The γ/δ T cells produced cytokines in response to adenovirus-peptide-loaded target cells, but not to control-peptide-loaded target cells (Fig. 5). The TCR-transfected CD8^+^ T cells produced lower amounts of IFNγ compared to TCR-transfected γ/δ T cells on colo829 and lower amounts of TNF on both targets (Fig. 5). Intracellular cytokine staining showed that only CD8^+^ T cells and γ/δ T cells transfected with the HAdV/A1-specific TCR recognized target cells loaded with the HAdV peptide and produced cytokines (Fig. 6). In average 32% of the transfected CD8^+^ T cells and 31% of the transfected γ/δ T cells specifically produced IFNγ, indicating that a higher percentage of the T cells was functionally transfected with the TCR, than one could have concluded from the streptamer-staining (Fig. 4).

Taken together, the co-transfection of CD8α and the HAdV/A1-specific TCR into γ/δ T cells led to an efficient antigen-specific cytokine production, and suggested that these cells might even have an increased functionality compared to HAdV/A1-TCR-transfected CD8^+^ T cells.

### Both HAdV/A1-TCR-transfected CD8^+^ T cells and γ/δ T cells lyse adenovirus-infected cells efficiently

**Figure 7:**
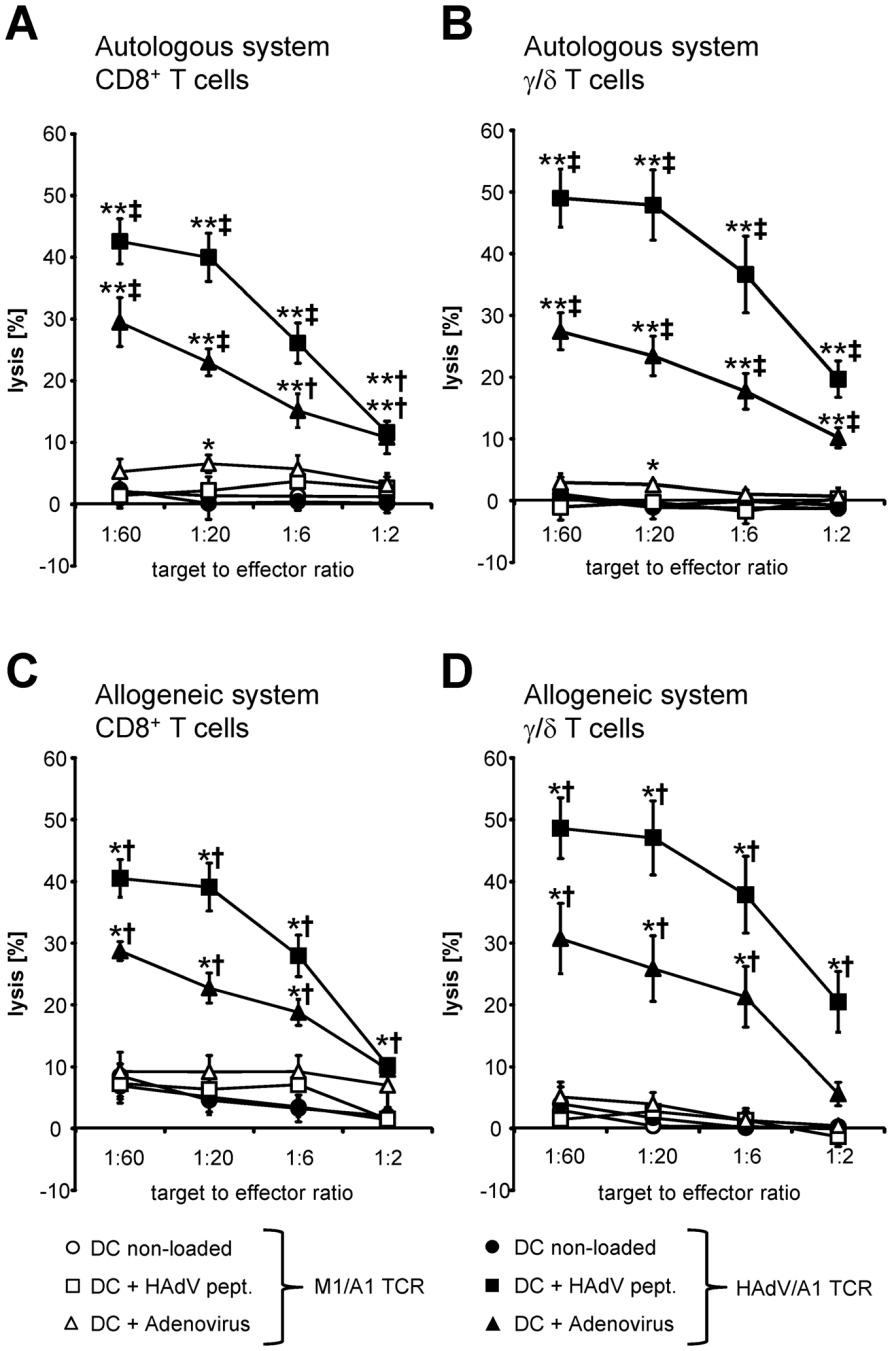
Cytolytic capacity of HAdV/A1-TCR-transfected CD8^+^ T cells and γ/δ T cells against adenovirus-infected target cells. CD8^+^ T cells (**A** and **C**) and γ/δ T cells (**B** and **D**) of the same donors were either electroporated with mRNA encoding the MAGE-1/A1-specific TCR (open symbols) or electroporated with mRNA encoding the HAdV/A1-specific TCR (closed symbols) either in combination with CD8α mRNA (γ/δ T cells) or not (CD8^+^ T cells). These cells were used as effector cells in standard 4–6 h cytotoxicity assays. Autologous (**A** and **B**) and allogenic (**C** and **D**) DC either untreated (non-loaded), loaded with the adenovirus peptide (Adeno pept.), or infected with adenovirus (adenovirus) were used as target cells and the percentage of lysed cells was calculated. The target to effector cell ratios were 1∶60, 1∶20, 1∶6, and 1∶2. Average values of 6 (**A** and **B**) or 4 (**C** and **D**) individual experiments (each performed in triplicates) +/− SEM are shown. P-values were calculated by the Mann-Whitney U test. For statistics, conditions were compared to the non-loaded DC condition (* and **) and to T cells transfected with the M1/A1 control TCR (^‡^ and ^†^). (** or ^‡^ p≤0.01; *or ^†^ p≤0.05). Raw data are summarized in Table S7A to D in File S1.

In our intended clinical setting, the elimination of virus-infected target cells will be one of the most important functions of the TCR-transfected T cells. Hence, the lytic capacity of HAdV/A1-TCR-transfected CD8^+^ T cells and HAdV/A1-TCR/CD8α co-transfected γ/δ T cells against adenovirus-infected DC was investigated. CD8^+^ T cells or γ/δ T cells transfected with a control TCR specific for MAGE-1 presented by HLA-A1 (M1/A1) induced no lysis of untreated DC and weak lysis of adenovirus-peptide-loaded and adenovirus-infected DC (Fig. 7A and B). HAdV/A1-TCR-transfected CD8^+^ T cells and HAdV/A1-TCR/CD8α co-transfected γ/δ T cells lysed adenovirus-peptide-loaded target cells very efficiently, while untreated target cells were not lysed (Fig. 7A and B). Most importantly, HAdV/A1-TCR-transfected CD8^+^ T cells and HAdV/A1-TCR/CD8α co-transfected γ/δ T cells were both able to lyse adenovirus-infected target cells antigen-specifically, although to a lesser extent than peptide-loaded target cells (Fig. 7A and B).

To address the point of allo-reactivity, we investigated the lytic capacity of allogeneic TCR-transfected CD8^+^ T cells and TCR/CD8-co-transfected γ/δ T cells on peptide-loaded and adenovirus-infected DC. As shown in figure 7C and D, the antigen-specific lysis was similar to that of the autologous system, and only a small increase in the background lysis of untreated DC by allogeneic CD8^+^ T cells compared to autologous CD8^+^ T cells was observed (compare to Fig. 7A and B). When comparing the background lysis by TCR-transfected CD8^+^ T cells and TCR/CD8-co-transfected γ/δ T cells in the allogeneic system, a slightly increased lysis induced by the former was seen.

Taken together, here we show, for the first time, that not only α/β T cells but also γ/δ T cells can be equipped with a HAdV specificity by TCR-RNA electroporation and that these cells are able to lyse adenovirus-infected target cells highly significantly.

## Discussion

In the current study we developed a novel therapeutic option for patients suffering from HAdV infection after allogeneic HSCT. It is based on the generation of HAdV-specific T cells from HAdV-seronegative donors by TCR-RNA electroporation, and it might present a promising alternative for other recently described procedures, like the cost-intensive and laborious long-term (10–14 weeks) *in vitro* expansion of HAdV-specific T cells from partially HLA-matched third-party donors, which are effective without the induction of severe GvHD as shown in a few clinical trials [36]–[38]. Another method reported the *in vitro* generation of HAdV-specific T cells from naïve T-cell populations in cord blood [39], which is even more costly and time-consuming. This holds also true for the recently described application of third-party “off the shelf” long-term expanded HAdV-specific T-cell lines, which showed impressive clinical results [40].

Quite recently, the use of TCR-transduced CMV-specific T cells has also been discussed to be a viable therapeutic option for patients after HSCT or solid organ transplantation (SOT) in case of sero-negative donors [18]. Although several TCR specific for CMV [23], [41]–[43] and EBV [44] are known, none were known for HAdV. We were the first to clone a HAdV-specific TCR sequence recognizing the immunodominant HLA-type A*0101-restricted viral 10-mer epitope LTDLGQNLLY (LTD) from the hexon protein of HAdV-species C, the predominant species in patients after HSCT [1]. These LTD-derived HAdV-specific T cells are also cross-reactive against several other HAdV species [6], which would enable a broad coverage of different species. According to data provided by www.allelefrequencies.net the HLA-type A*0101 is highly frequent and covers about 30% of the White population. Therefore, approximately 30% of all pediatric patients could be potential candidates for this kind of treatment. The coverage could be even increased to 72% if three additional TCRs against the already known and immunodominant peptide-MHC class I complexes for the HLA-types A*2401, B*0702, and B*3501 [6] would be identified and used for transfer to T cells.

The feasibility of mRNA electroporation to transfer TCR to bulk T cells has already been described for tumor-specific [31], [45], HIV-1-specific [34], and CMV-specific [43] TCR. Although receptor expression via mRNA electroporation is transient, effector functions such as cytokine production and lytic activity can still be measured for at least 3 days post electroporation [19], [20], [31], [46]. Complete loss of receptor expression and redirected specificity was seen 9 days after electroporation [19]. Because the period of complete immunosuppression after HSCT is limited, and only a temporary exogenous defense against the virus is required, we consider the mRNA-electroporation technology well suited to be applied here. All together, these results support the assumption that there is sufficient time for TCR-RNA-electroporated T cells to fulfill their task before losing their specificity, provided that multiple injections over the period of complete immune suppression are performed.

Although the time-limited TCR expression is a disadvantage compared to retroviral transduction, mRNA electroporation is a much safer method [31]. In contrast to lentiviral transduction, mRNA electroporation is faster and allows for high numbers of modified T cells due to high transfection rates (>80%) [20]. However, infusion of high doses of mRNA-electroporated T cells, which still retain their allo-reactive potential, highly increases the risk for GvHD in patients after allogeneic HSCT and is only suitable for HLA-matched related donors. Therefore, we used γ/δ T cells, which are known to be involved in viral defense after HSCT without the potential to induce GvHD in patients [22], instead of α/β T cells. Furthermore, preliminary results of adoptive immunotherapy using γ/δ T cells showed promising results in clinical trials [47], [48]. Moreover, γ/δ T cells were isolated and expanded to sufficient numbers for clinical application by several groups [49]–[52]. An additional risk that is overcome by the use of γ/δ T cells, is the problem of TCR-chain mispairing, as it has been shown that introduced TCR α- and β-chains can mispair with their endogenous counterparts [41]. This could result, in theory, in the formation of new, auto-reactive receptors. Although the resulting danger of autoimmunity is already reduced even in α/β T cells by the transiency of the mRNA transfection, it is further reduced by the use of γ/δ T cells because TCR α/β-chains preferentially pair with each other instead of forming heterodimers with γ/δ-chains [53], [54].

Although our isolated γ/δ T cells lacked the co-stimulatory molecule CD8α, HAdV/A1-specific TCR-redirected γ/δ T cells were able to produce cytokines after stimulation with adenovirus-peptide-loaded target cells. Nevertheless, the cytokine production was highly increased after co-transfection with the co-stimulatory molecule CD8α. This is in accordance with the data shown in Figure 1, where the signal strength benefited from the presence of CD8. These data provide evidence that the functionality of the HAdV/A1-specific TCR is still obvious without CD8α but even higher when combined with CD8α transfection. Similar results have been obtained by Van der Veken et al. [24]. Notwithstanding, the combined transfection of the HAdV/A1-specific TCR and CD8α can be easily implemented into a clinical-based protocol.

Strikingly, both HAdV/A1-TCR-transfected primary CD8^+^ α/β T cells and HAdV-TCR/CD8α co-transfected γ/δ T cells lysed adenovirus-infected target cells (Fig. 7). Moreover, co-transfection of γ/δ T cells with the HAdV/A1-TCR and CD8α seems to be a feasible method to generate not allo-reactive T cells with strong antiviral activity. In fact, we expected to see an allo-reactivity of CD8^+^ T cells (independent of the TCR introduced) against allogeneic DC. The observed weakness of this allo-reactivity could have been caused by the limited duration of the assay. Elongating the incubation time in the allogeneic system could show a clearer difference in allo-reactive capacity between γ/δ T cells and CD8^+^ α/β T cells.

Taken together, this is the first report identifying the coding sequence of a HAdV-specific TCR, which we then functionally transferred to α/β T cells and γ/δ T cells by RNA electroporation. Because of the high frequency of HLA-type A*0101 in the White population, about 30% of pediatric patients might benefit from treatment with TCR-transfected γ/δ T cells. Although the use of third-party HAdV-specific T-cell lines is a promising option to treat patients whose original HSCT-donors have no detectable HAdV-specific T-cells, the use of TCR-transfected γ/δ T cells could be a promising alternative. It should further reduce the risk for GvHD, the costs, and laborious expansion protocols (2 weeks instead of 10–14 weeks).

We strongly believe that especially the use of TCR/CD8α-co-transfected γ/δ T cells offers a new means for the immunotherapy of HAdV-infected patients after allogeneic HSCT.

## Supporting Information

File S1 **Combined file of supporting tables.** Table S1: raw data to Fig. 1. Table S2A: raw data to Fig. 2A. Table S2B: raw data to Fig. 2B. Table S3A: raw data to Fig. 3A. Table S3B: raw data to Fig. 3B. Table S4: raw data to Fig. 4. Table S5A: raw data to Fig. 5A. Table S5B: raw data to Fig. 5B. Table S6: raw data to Fig. 6. Table S7A: raw data to Fig. 7A. Table S7B: raw data to Fig. 7B. Table S7C: raw data to Fig. 7C. Table S7D: raw data to Fig. 7D.
(PDF)

## References

[1] Eiz-Vesper B, Maecker-Kolhoff B, Blasczyk R (2012) Adoptive T-cell immunotherapy from third-party donors: characterization of donors and set up of a T-cell donor registry. Front Immunol 3: 410 10.3389/fimmu.2012.00410 [doi] 23372567 10.3389/fimmu.2012.00410 PMC3556568

[2] Matthes-Martin S, Feuchtinger T, Shaw PJ, Engelhard D, Hirsch HH, et al (2012) European guidelines for diagnosis and treatment of adenovirus infection in leukemia and stem cell transplantation: summary of ECIL-4 (2011). Transpl Infect Dis 14: 555–563 10.1111/tid.12022 [doi] 23146063 10.1111/tid.12022

[3] Symeonidis N, Jakubowski A, Pierre-Louis S, Jaffe D, Pamer E, et al (2007) Invasive adenoviral infections in T-cell-depleted allogeneic hematopoietic stem cell transplantation: high mortality in the era of cidofovir. Transpl Infect Dis 9: 108–113 TID184 [pii];10.1111/j.1399-3062.2006.00184.x [doi] 17461995 10.1111/j.1399-3062.2006.00184.x

[4] Feuchtinger T, Matthes-Martin S, Richard C, Lion T, Fuhrer M, et al (2006) Safe adoptive transfer of virus-specific T-cell immunity for the treatment of systemic adenovirus infection after allogeneic stem cell transplantation. Br J Haematol 134: 64–76 BJH6108 [pii];10.1111/j.1365-2141.2006.06108.x [doi] 16803570 10.1111/j.1365-2141.2006.06108.x

[5] Leen AM, Christin A, Myers GD, Liu H, Cruz CR, et al (2009) Cytotoxic T lymphocyte therapy with donor T cells prevents and treats adenovirus and Epstein-Barr virus infections after haploidentical and matched unrelated stem cell transplantation. Blood 114: 4283–4292 blood-2009-07-232454 [pii];10.1182/blood-2009-07-232454 [doi] 19700662 10.1182/blood-2009-07-232454 PMC2774556

[6] Geyeregger R, Freimuller C, Stevanovic S, Stemberger J, Mester G, et al (2013) Short-term in-vitro expansion improves monitoring and allows affordable generation of virus-specific T-cells against several viruses for a broad clinical application. PLoS One 8: e59592 10.1371/journal.pone.0059592 [doi];PONE-D-12-34959 [pii] 23630567 10.1371/journal.pone.0059592 PMC3632539

[7] Geyeregger R, Freimuller C, Stemberger J, Artwohl M, Witt V, et al (2014) First-in-man clinical results with good manufacturing practice (GMP)-compliant polypeptide-expanded adenovirus-specific T cells after haploidentical hematopoietic stem cell transplantation. J Immunother 37: 245–249 10.1097/CJI.0000000000000034 [doi];00002371-201405000-00006 [pii] 24714358 10.1097/CJI.0000000000000034

[8] Leen AM, Sili U, Vanin EF, Jewell AM, Xie W, et al (2004) Conserved CTL epitopes on the adenovirus hexon protein expand subgroup cross-reactive and subgroup-specific CD8+ T cells. Blood 104: 2432–2440 10.1182/blood-2004-02-0646 [doi];2004-02-0646 [pii] 15265797 10.1182/blood-2004-02-0646

[9] Garnett CT, Erdman D, Xu W, Gooding LR (2002) Prevalence and quantitation of species C adenovirus DNA in human mucosal lymphocytes. J Virol 76: 10608–10616. 12368303 10.1128/JVI.76.21.10608-10616.2002 PMC136639

[10] Pichla-Gollon SL, Drinker M, Zhou X, Xue F, Rux JJ, et al (2007) Structure-based identification of a major neutralizing site in an adenovirus hexon. J Virol 81: 1680–1689 JVI.02023-06 [pii];10.1128/JVI.02023-06 [doi] 17108028 10.1128/JVI.02023-06 PMC1797575

[11] Thorner AR, Vogels R, Kaspers J, Weverling GJ, Holterman L, et al (2006) Age dependence of adenovirus-specific neutralizing antibody titers in individuals from sub-Saharan Africa. J Clin Microbiol 44: 3781–3783 44/10/3781 [pii];10.1128/JCM.01249-06 [doi] 17021110 10.1128/JCM.01249-06 PMC1594810

[12] Sun C, Zhang Y, Feng L, Pan W, Zhang M, et al (2011) Epidemiology of adenovirus type 5 neutralizing antibodies in healthy people and AIDS patients in Guangzhou, southern China. Vaccine 29: 3837–3841 S0264-410X(11)00410-5 [pii];10.1016/j.vaccine.2011.03.042 [doi] 21447314 10.1016/j.vaccine.2011.03.042

[13] Park TS, Rosenberg SA, Morgan RA (2011) Treating cancer with genetically engineered T cells. Trends Biotechnol 29: 550–557 S0167-7799(11)00075-8 [pii];10.1016/j.tibtech.2011.04.009 [doi] 21663987 10.1016/j.tibtech.2011.04.009 PMC3193849

[14] Kessels HW, Wolkers MC, van den Boom MD, van der Valk MA, Schumacher TN (2001) Immunotherapy through TCR gene transfer. Nat Immunol 2: 957–961 10.1038/ni1001-957 [doi];ni1001-957 [pii] 11577349 10.1038/ni1001-957

[15] Morris EC, Tsallios A, Bendle GM, Xue SA, Stauss HJ (2005) A critical role of T cell antigen receptor-transduced MHC class I-restricted helper T cells in tumor protection. Proc Natl Acad Sci U S A 102: 7934–7939 0500357102 [pii];10.1073/pnas.0500357102 [doi] 15908507 10.1073/pnas.0500357102 PMC1142362

[16] Stanislawski T, Voss RH, Lotz C, Sadovnikova E, Willemsen RA, et al (2001) Circumventing tolerance to a human MDM2-derived tumor antigen by TCR gene transfer. Nat Immunol 2: 962–970. 11577350 10.1038/ni1001-962

[17] Morgan RA, Dudley ME, Wunderlich JR, Hughes MS, Yang JC, et al (2006) Cancer regression in patients after transfer of genetically engineered lymphocytes. Science 314: 126–129. 16946036 10.1126/science.1129003 PMC2267026

[18] Schub A, Schuster IG, Hammerschmidt W, Moosmann A (2009) CMV-specific TCR-transgenic T cells for immunotherapy. J Immunol 183: 6819–6830 jimmunol.0902233 [pii];10.4049/jimmunol.0902233 [doi] 19864595 10.4049/jimmunol.0902233

[19] Birkholz K, Hombach A, Krug C, Reuter S, Kershaw M, et al (2009) Transfer of mRNA encoding recombinant immunoreceptors reprograms CD4+ and CD8+ T cells for use in the adoptive immunotherapy of cancer. Gene Ther 16: 596–604. 19158846 10.1038/gt.2008.189

[20] Koh S, Shimasaki N, Suwanarusk R, Ho ZZ, Chia A, et al (2013) A practical approach to immunotherapy of hepatocellular carcinoma using T cells redirected against hepatitis B virus. Mol Ther Nucleic Acids 2: e114 mtna201343 [pii];10.1038/mtna.2013.43 [doi] 23941866 10.1038/mtna.2013.43 PMC3759740

[21] Breuer S, Rauch M, Matthes-Martin S, Lion T (2012) Molecular diagnosis and management of viral infections in hematopoietic stem cell transplant recipients. Mol Diagn Ther 16: 63–77 1 [pii];10.2165/11631490-000000000-00000 [doi] 22497528 10.1007/BF03256431

[22] Oevermann L, Lang P, Feuchtinger T, Schumm M, Teltschik HM, et al (2012) Immune reconstitution and strategies for rebuilding the immune system after haploidentical stem cell transplantation. Ann N Y Acad Sci 1266: 161–170 10.1111/j.1749-6632.2012.06606.x [doi] 22901267 10.1111/j.1749-6632.2012.06606.x

[23] van der Veken LT, Hagedoorn RS, van Loenen MM, Willemze R, Falkenburg JH, et al (2006) Alphabeta T-cell receptor engineered gammadelta T cells mediate effective antileukemic reactivity. Cancer Res 66: 3331–3337 66/6/3331 [pii];10.1158/0008-5472.CAN-05-4190 [doi] 16540688 10.1158/0008-5472.CAN-05-4190

[24] van der Veken LT, Coccoris M, Swart E, Falkenburg JH, Schumacher TN, et al. (2009) Alpha beta T cell receptor transfer to gamma delta T cells generates functional effector cells without mixed TCR dimers in vivo. J Immunol 182: 164–170. 182/1/164 [pii]. 10.4049/jimmunol.182.1.164 19109147

[25] Hanagiri T, Shigematsu Y, Kuroda K, Baba T, Shiota H, et al (2012) Antitumor activity of human gammadelta T cells transducted with CD8 and with T-cell receptors of tumor-specific cytotoxic T lymphocytes. Cancer Sci 103: 1414–1419 10.1111/j.1349-7006.2012.02337.x [doi] 22621620 10.1111/j.1349-7006.2012.02337.x PMC7659244

[26] Krug C, Wiesinger M, Abken H, Schuler-Thurner B, Schuler G, et al. (2014) A GMP-compliant protocol to expand and transfect cancer patient T cells with mRNA encoding a tumor-specific chimeric antigen receptor. Cancer Immunol Immunother. 10.1007/s00262-014-1572-5 [doi]. 10.1007/s00262-014-1572-5 PMC11029092 24938475

[27] Dorrie J, Schaft N, Muller I, Wellner V, Schunder T, et al (2008) Introduction of functional chimeric E/L-selectin by RNA electroporation to target dendritic cells from blood to lymph nodes. Cancer Immunol Immunother 57: 467–477. 17768622 10.1007/s00262-007-0385-1 PMC11041385

[28] Felzmann T, Buchberger M, Jechlinger M, Kircheis R, Wagner E, et al. (2000) Xenogenization by tetanus toxoid loading into lymphoblastoid cell lines and primary human tumor cells mediated by polycations and liposomes. Cancer Lett 161: 241–250. S0304383500006182 [pii]. 10.1016/s0304-3835(00)00618-2 11090975

[29] Birkholz K, Hofmann C, Hoyer S, Schulz B, Harrer T, et al (2009) A fast and robust method to clone and functionally validate T-cell receptors. J Immunol Methods 346: 45–54. 19427315 10.1016/j.jim.2009.05.001

[30] Arden B, Clark SP, Kabelitz D, Mak TW (1995) Human T-cell receptor variable gene segment families. Immunogenetics 42: 455–500. 8550092 10.1007/BF00172176

[31] Schaft N, Dorrie J, Muller I, Beck V, Baumann S, et al (2006) A new way to generate cytolytic tumor-specific T cells: electroporation of RNA coding for a T cell receptor into T lymphocytes. Cancer Immunol Immunother 55: 1132–1141. 16344988 10.1007/s00262-005-0098-2 PMC11030166

[32] Knippertz I, Hesse A, Schunder T, Kampgen E, Brenner MK, et al (2009) Generation of human dendritic cells that simultaneously secrete IL-12 and have migratory capacity by adenoviral gene transfer of hCD40L in combination with IFN-gamma. J Immunother 32: 524–538. 19609245 10.1097/CJI.0b013e3181a28422

[33] Schierer S, Hesse A, Muller I, Kampgen E, Curiel DT, et al (2008) Modulation of viability and maturation of human monocyte-derived dendritic cells by oncolytic adenoviruses. Int J Cancer 122: 219–229 10.1002/ijc.23074 [doi] 17764070 10.1002/ijc.23074

[34] Hofmann C, Harrer T, Kubesch V, Maurer K, Metzner KJ, et al (2008) Generation of HIV-1-specific T cells by electroporation of T-cell receptor RNA. AIDS 22: 1577–1582. 18670216 10.1097/QAD.0b013e3283063a17

[35] Hoyer S, Prommersberger S, Pfeiffer IA, Schuler-Thurner B, Schuler G, et al. (2014) Concurrent interaction of DCs with CD4 and CD8 T cells improves secondary CTL expansion: It takes three to tango. Eur J Immunol. 10.1002/eji.201444477 [doi]. 10.1002/eji.201444477 25211552

[36] Qasim W, Derniame S, Gilmour K, Chiesa R, Weber M, et al (2011) Third-party virus-specific T cells eradicate adenoviraemia but trigger bystander graft-versus-host disease. Br J Haematol 154: 150–153 10.1111/j.1365-2141.2011.08579.x [doi] 21501134 10.1111/j.1365-2141.2011.08579.x

[37] Feuchtinger T, Opherk K, Bethge WA, Topp MS, Schuster FR, et al (2010) Adoptive transfer of pp65-specific T cells for the treatment of chemorefractory cytomegalovirus disease or reactivation after haploidentical and matched unrelated stem cell transplantation. Blood 116: 4360–4367 blood-2010-01-262089 [pii];10.1182/blood-2010-01-262089 [doi] 20625005 10.1182/blood-2010-01-262089

[38] Uhlin M, Gertow J, Uzunel M, Okas M, Berglund S, et al (2012) Rapid salvage treatment with virus-specific T cells for therapy-resistant disease. Clin Infect Dis 55: 1064–1073 cis625 [pii];10.1093/cid/cis625 [doi] 22806594 10.1093/cid/cis625

[39] Hanley PJ, Cruz CR, Savoldo B, Leen AM, Stanojevic M, et al (2009) Functionally active virus-specific T cells that target CMV, adenovirus, and EBV can be expanded from naive T-cell populations in cord blood and will target a range of viral epitopes. Blood 114: 1958–1967 blood-2009-03-213256 [pii];10.1182/blood-2009-03-213256 [doi] 19443656 10.1182/blood-2009-03-213256 PMC2738578

[40] Leen AM, Bollard CM, Mendizabal AM, Shpall EJ, Szabolcs P, et al (2013) Multicenter study of banked third-party virus-specific T cells to treat severe viral infections after hematopoietic stem cell transplantation. Blood 121: 5113–5123 blood-2013-02-486324 [pii];10.1182/blood-2013-02-486324 [doi] 23610374 10.1182/blood-2013-02-486324 PMC3695359

[41] Heemskerk MH, Hagedoorn RS, van der Hoorn MA, van der Veken LT, Hoogeboom M, et al (2007) Efficiency of T-cell receptor expression in dual-specific T cells is controlled by the intrinsic qualities of the TCR chains within the TCR-CD3 complex. Blood 109: 235–243. 16968899 10.1182/blood-2006-03-013318

[42] van Lent AU, Nagasawa M, van Loenen MM, Schotte R, Schumacher TN, et al. (2007) Functional human antigen-specific T cells produced in vitro using retroviral T cell receptor transfer into hematopoietic progenitors. J Immunol 179: 4959–4968. 179/8/4959 [pii]. 10.4049/jimmunol.179.8.4959 17911580

[43] Thomas S, Klobuch S, Besold K, Plachter B, Dorrie J, et al (2012) Strong and sustained effector function of memory- versus naive-derived T cells upon T-cell receptor RNA transfer: implications for cellular therapy. Eur J Immunol 42: 3442–3453 10.1002/eji.201242666 [doi] 22930221 10.1002/eji.201242666

[44] Schaft N, Lankiewicz B, Drexhage J, Berrevoets C, Moss DJ, et al (2006) T cell re-targeting to EBV antigens following TCR gene transfer: CD28-containing receptors mediate enhanced antigen-specific IFNgamma production. Int Immunol 18: 591–601. 16507598 10.1093/intimm/dxh401

[45] Zhao Y, Zheng Z, Robbins PF, Khong HT, Rosenberg SA, et al (2005) Primary human lymphocytes transduced with NY-ESO-1 antigen-specific TCR genes recognize and kill diverse human tumor cell lines. J Immunol 174: 4415–4423. 15778407 10.4049/jimmunol.174.7.4415 PMC2174604

[46] Lehner M, Gotz G, Proff J, Schaft N, Dorrie J, et al (2012) Redirecting T cells to Ewing's sarcoma family of tumors by a chimeric NKG2D receptor expressed by lentiviral transduction or mRNA transfection. PLoS One 7: e31210. 22355347 10.1371/journal.pone.0031210 PMC3280271

[47] Kobayashi H, Tanaka Y, Shimmura H, Minato N, Tanabe K (2010) Complete remission of lung metastasis following adoptive immunotherapy using activated autologous gammadelta T-cells in a patient with renal cell carcinoma. Anticancer Res 30: 575–579. 30/2/575 [pii]. 20332473

[48] Nakajima J, Murakawa T, Fukami T, Goto S, Kaneko T, et al (2010) A phase I study of adoptive immunotherapy for recurrent non-small-cell lung cancer patients with autologous gammadelta T cells. Eur J Cardiothorac Surg 37: 1191–1197 S1010-7940(09)01149-X [pii];10.1016/j.ejcts.2009.11.051 [doi] 20137969 10.1016/j.ejcts.2009.11.051

[49] Salot S, Bercegeay S, Dreno B, Saiagh S, Scaglione V, et al (2009) Large scale expansion of Vgamma9Vdelta2 T lymphocytes from human peripheral blood mononuclear cells after a positive selection using MACS “TCR gamma/delta+ T cell isolation kit”. J Immunol Methods 347: 12–18 S0022-1759(09)00154-9 [pii];10.1016/j.jim.2009.05.006 [doi] 19465023 10.1016/j.jim.2009.05.006

[50] Dokouhaki P, Han M, Joe B, Li M, Johnston MR, et al (2010) Adoptive immunotherapy of cancer using ex vivo expanded human gammadelta T cells: A new approach. Cancer Lett 297: 126–136 S0304-3835(10)00258-2 [pii];10.1016/j.canlet.2010.05.005 [doi] 20537791 10.1016/j.canlet.2010.05.005

[51] Tsuda J, Li W, Yamanishi H, Yamamoto H, Okuda A, et al (2011) Involvement of CD56brightCD11c+ cells in IL-18-mediated expansion of human gammadelta T cells. J Immunol 186: 2003–2012 jimmunol.1001919 [pii];10.4049/jimmunol.1001919 [doi] 21239711 10.4049/jimmunol.1001919

[52] Siegers GM, Ribot EJ, Keating A, Foster PJ (2013) Extensive expansion of primary human gamma delta T cells generates cytotoxic effector memory cells that can be labeled with Feraheme for cellular MRI. Cancer Immunol Immunother 62: 571–583 10.1007/s00262-012-1353-y [doi] 23100099 10.1007/s00262-012-1353-y PMC11029191

[53] Koning F, Maloy WL, Cohen D, Coligan JE (1987) Independent association of T cell receptor beta and gamma chains with CD3 in the same cell. J Exp Med 166: 595–600. 3110356 10.1084/jem.166.2.595 PMC2189592

[54] Saito T, Hochstenbach F, Marusic-Galesic S, Kruisbeek AM, Brenner M, et al (1988) Surface expression of only gamma delta and/or alpha beta T cell receptor heterodimers by cells with four (alpha, beta, gamma, delta) functional receptor chains. J Exp Med 168: 1003–1020. 2971751 10.1084/jem.168.3.1003 PMC2189041

