# Supplementary material for: Human Adenovirus-Specific γ/δ and CD8+ T Cells Generated by T-Cell Receptor Transfection to Treat Adenovirus Infection after Allogeneic Stem Cell Transplantation
Source: PLoS One. 2014 Oct 7;9(10):e109944. doi: 10.1371/journal.pone.0109944 (PMC4188623; doi:10.1371/journal.pone.0109944)
Supplement: File S1 — Combined file of supporting tables. Table S1: raw data to Fig. 1. Table S2A: raw data to Fig. 2A. Table S2B: raw data to Fig. 2B. Table S3A: raw data to Fig. 3A. Table S3B: raw data to Fig. 3B. Table S4: raw data to Fig. 4. Table S5A: raw data to Fig. 5A. Table S5B: raw data to Fig. 5B. Table S6: raw data to Fig. 6. Table S7A: raw data to Fig. 7A. Table S7B: raw data to Fig. 7B. Table S7C: raw data to Fig. 7C. Table S7D: raw data to Fig. 7D. (PDF) [file pone.0109944.s001.pdf]

Table S1: raw data to Fig. 1

Light units

	Exp. 1	Exp. 2	Exp. 3	Exp. 4	Exp. 5
CD4 Jurkat Adeno TCR w/o peptide	697,00		296,00	215,00	248,00
CD4 Jurkat Adeno TCR Adeno/A1 peptide	2764,00		702,00	902,00	603,00
CD4 Jurkat Adeno TCR MAGE3/A1 peptide	337,00		237,00	239,00	253,00
CD4 Jurkat MAGE-A3 TCR w/o peptide	1198,00		365,00	302,00	343,00
CD4 Jurkat MAGE-A3 TCR Adeno/A1 peptide			351,00	350,00	344,00
CD4 Jurkat MAGE-A3 TCR MAGE3/A1 peptide	1280,00		350,00	343,00	302,00
CD8 Jurkat Adeno TCR w/o peptide	1479,00	186,00	2090,00	1820,00	1819,00
CD8 Jurkat Adeno TCR Adeno/A1 peptide	19830,00	2515,00	27128,00	24010,00	23518,00
CD8 Jurkat Adeno TCR MAGE3/A1 peptide	1464,00	273,00	1666,00	1465,00	1976,00
CD8 Jurkat MAGE-A3 TCR w/o peptide	1944,00	515,00	2406,00	1810,00	2731,00
CD8 Jurkat MAGE-A3 TCR Adeno/A1 peptide		266,00	2298,00	2498,00	2203,00
CD8 Jurkat MAGE-A3 TCR MAGE3/A1 peptide	25692,00	2796,00	20618,00	20907,00	18207,00

Fold induction

	Exp. 1	Exp. 2	Exp. 3	Exp. 4	Exp. 5
CD4 Jurkat Adeno TCR w/o peptide	1,00		1,00	1,00	1,00
CD4 Jurkat Adeno TCR Adeno/A1 peptide	3,97		2,37	4,20	2,43
CD4 Jurkat Adeno TCR MAGE3/A1 peptide	0,48		0,80	1,11	1,02
CD4 Jurkat MAGE-A3 TCR w/o peptide	1,00		1,00	1,00	1,00
CD4 Jurkat MAGE-A3 TCR Adeno/A1 peptide			0,96	1,16	1,00
CD4 Jurkat MAGE-A3 TCR MAGE3/A1 peptide	1,07		0,96	1,14	0,88
CD8 Jurkat Adeno TCR w/o peptide	1,00	1,00	1,00	1,00	1,00
CD8 Jurkat Adeno TCR Adeno/A1 peptide	13,41	13,52	12,98	13,19	12,93
CD8 Jurkat Adeno TCR MAGE3/A1 peptide	0,99	1,47	0,80	0,80	1,09
CD8 Jurkat MAGE-A3 TCR w/o peptide	1,00	1,00	1,00	1,00	1,00
CD8 Jurkat MAGE-A3 TCR Adeno/A1 peptide		0,52	0,96	1,38	0,81
CD8 Jurkat MAGE-A3 TCR MAGE3/A1 peptide	13,22	5,43	8,57	11,55	6,67

Table S2A: raw data to Fig. 2A

IL-2 (pg/ml)	Experiment 1	Experiment 2	Experiment 3	Experiment 4	Experiment 5
mock w/o peptide	9,44	0,87	0,55	1,76	0,60
mock Adeno/A1 peptide	40,95	4,93	6,46	19,26	1,54
Adeno TCR w/o peptide	8,79	0,77	0,33	2,01	1,24
Adeno TCR Adeno/A1 peptide	5366,83	2695,51	512,54	2197,97	3528,27

TNF (pg/ml)	Experiment 1	Experiment 2	Experiment 3	Experiment 4	Experiment 5
mock w/o peptide	11,37	5,45	10,28	4,33	15,29
mock Adeno/A1 peptide	14,50	7,51	34,90	5,49	26,46
Adeno TCR w/o peptide	11,23	5,85	7,96	7,55	16,40
Adeno TCR Adeno/A1 peptide	3369,04	1870,31	629,67	258,98	1312,59

IFNgamma (pg/ml)	Experiment 1	Experiment 2	Experiment 3	Experiment 4	Experiment 5
mock w/o peptide	13,51	4,73	3,69	5,85	2,67
mock Adeno/A1 peptide	79,31	32,93	77,73	66,03	3,75
Adeno TCR w/o peptide	27,71	3,30	6,50	4,57	0,00
Adeno TCR Adeno/A1 peptide	10717,56	5861,46	732,28	8113,20	5798,47

Table S2B: raw data to Fig. 2B

IL-2 (pg/ml)	Exp. 1	Exp. 2	Exp. 3	Exp. 4	Exp. 5	Exp. 6	Exp. 7
mock w/o virus	0,40	0,60	0,97	1,76	9,44	0,87	0,55
mock w/ virus	109,31	3,40	0,91	109,89	196,53	31,75	10,56
Adeno TCR w/o virus	2,35	1,24	5,00	2,01	8,79	0,77	0,33
Adeno TCR w/ virus	2158,86	1100,94	469,21	1587,89	4994,01	1340,91	824,26

TNF (pg/ml)	Exp. 1	Exp. 2	Exp. 3	Exp. 4	Exp. 5	Exp. 6	Exp. 7
mock w/o virus	2,54	15,29	2,55	4,33	11,37	5,45	10,28
mock w/ virus	3,82	12,98	3,51	7,35	36,19	5,75	60,84
Adeno TCR w/o virus	1,98	16,40	3,19	7,55	11,23	5,85	7,96
Adeno TCR w/ virus	134,55	342,22	38,11	157,62	2968,48	854,08	969,22

IFNgamma (pg/ml)	Exp. 1	Exp. 2	Exp. 3	Exp. 4	Exp. 5	Exp. 6	Exp. 7
mock w/o virus	2,83	2,67	11,60	5,85	13,51	4,73	3,69
mock w/ virus	138,34	5,42	9,88	310,28	449,54	91,44	161,79
Adeno TCR w/o virus	4,52	0,00	96,36	4,57	27,71	3,30	6,50
Adeno TCR w/ virus	2927,94	1420,44	2354,34	7932,33	8951,21	4554,01	1575,21

Table S3A: raw data to Fig. 3A

IL-2 (pg/ml)	Experiment 1	Experiment 2	Experiment 3
mock w/o pept	1,04	0,65	1,37
mock w/ Adeno pept	1,10	0,60	1,86
Adeno TCR w/o pept	0,29	0,19	0,99
Adeno TCR w/ Adeno pept	0,99	1,15	1,48
Adeno TCR+CD8a w/o pept.	0,14	0,34	1,53
Adeno TCR+CD8a w/ Adeno pept.	23,04	5,97	70,66

TNF (pg/ml)	Experiment 1	Experiment 2	Experiment 3
mock w/o pept	10,42	4,03	13,74
mock w/ Adeno pept	3,77	3,35	14,78
Adeno TCR w/o pept	8,95	3,46	17,38
Adeno TCR w/ Adeno pept	35,39	3,87	156,97
Adeno TCR+CD8a w/o pept.	6,65	0,00	12,04
Adeno TCR+CD8a w/ Adeno pept.	435,11	26,86	1926,08

IFNgamma (pg/ml)	Experiment 1	Experiment 2	Experiment 3
mock w/o pept	12,58	50,40	2222,25
mock w/ Adeno pept	12,58	53,36	2818,75
Adeno TCR w/o pept	13,69	119,46	2263,28
Adeno TCR w/ Adeno pept	1183,99	677,74	6621,23
Adeno TCR+CD8a w/o pept.	19,10	167,49	1849,38
Adeno TCR+CD8a w/ Adeno pept.	10768,81	3205,58	20284,65

Table S3B : raw data to Fig. 3B

IL-2 (pg/ml)	Experiment 1	Experiment 2	Experiment 3
mock w/o pept	1,57	0,34	0,04
mock w/ Adeno pept	0,55	0,29	0,18
Adeno TCR w/o pept	1,10	0,19	0,38
Adeno TCR w/ Adeno pept	2,96	101,62	30,85
Adeno TCR+CD8a w/o pept.	1,31	0,00	0,04
Adeno TCR+CD8a w/ Adeno pept.	21,91	559,27	199,34

TNF (pg/ml)	Experiment 1	Experiment 2	Experiment 3
mock w/o pept	1,41	1,33	5,21
mock w/ Adeno pept	1,52	1,69	3,19
Adeno TCR w/o pept	0,73	1,48	6,90
Adeno TCR w/ Adeno pept	27,07	3486,66	1052,82
Adeno TCR+CD8a w/o pept.	1,99	4,09	5,21
Adeno TCR+CD8a w/ Adeno pept.	170,85	4959,13	1533,30

IFNgamma (pg/ml)	Experiment 1	Experiment 2	Experiment 3
mock w/o pept	5,25	6,16	85,21
mock w/ Adeno pept	3,70	0,22	89,84
Adeno TCR w/o pept	3,70	49,58	207,92
Adeno TCR w/ Adeno pept	1120,19	16442,33	16191,35
Adeno TCR+CD8a w/o pept.	3,40	21,17	153,99
Adeno TCR+CD8a w/ Adeno pept.	5324,20	19691,21	19922,25

Table S4: raw data to Fig. 4

CD8 T + MAGE1 TCR	Donor 1	Donor 2	Donor 3	Donor 4
gamma/delta FITC	8,12	2,65	4,37	3,29
CD4 FITC	1,22	0,7	0,79	1,06
CD8 FITC	95,71	93,06	81,64	92,44
CD16 FITC	-0,01	-0,02	0,17	1,72
CD19 FITC	0,05	0,13	0,07	0,07
CD14 FITC	2,85	1,2	2,95	2,07
Streptamer-PE	1,65	1,25	0,68	3,02

CD8 T + Adeno TCR	Donor 1	Donor 2	Donor 3	Donor 4
gamma/delta FITC	7,56	2,74	4,06	3,45
CD4 FITC	1,24	0,78	0,54	1,37
CD8 FITC	96,4	77,88	81,71	93,58
CD16 FITC	0,2	0,08	0,19	0,9
CD19 FITC	-0,09	-0,19	0,01	-0,11
CD14 FITC	2,98	1,35	2,62	1,81
Streptamer-PE	46,68	15,43	6,34	18,27

g/d T + MAGE1 TCR	Donor 1	Donor 2	Donor 3	Donor 4
gamma/delta FITC	96,34	96,35	85,56	94,44
CD4 FITC	2,2	1,08	0,22	0,63
CD8 FITC	71,13	39,3	33,37	48,33
CD16 FITC	0,63	0,94	1,99	24,88
CD19 FITC	-0,16	0,38	-0,05	0,1
CD14 FITC	5,82	0,52	4,9	3,59
Streptamer-PE	3,36	1,54	1,44	2,22

g/d T + Adeno TCR	Donor 1	Donor 2	Donor 3	Donor 4
gamma/delta FITC	96,11	93,57	80,65	94,27
CD4 FITC	2,11	0,82	0,2	0,52
CD8 FITC	77,25	49,48	43,79	55,04
CD16 FITC	0,5	0,47	1,82	22,6
CD19 FITC	-0,07	0,01	-0,03	0,06
CD14 FITC	4,73	3,57	5,79	2,76
Streptamer-PE	47,46	15,38	17,59	16,18

Table S5A : raw data to Fig. 5A

IL-2 (pg/ml)	Experiment 1	Experiment 2	Experiment 3
CD8 T +mock control	2,66	1,34	2,53
CD8 T +mock w/ Adeno pept	2,41	3,62	2,53
CD8 T +Adeno TCR control	2,03	1,58	6,03
CD8 T +Adeno TCR w/ Adeno pept	124,61	252,85	114,56
g/d +Adeno TCR+CD8a control	0,22	0,00	0,54
g/d +Adeno TCR+CD8a Adeno pept	13,36	559,27	134,88

TNF (pg/ml)	Experiment 1	Experiment 2	Experiment 3
CD8 T +mock control	1,13	3,27	3,71
CD8 T +mock w/ Adeno pept	2,96	2,56	1,57
CD8 T +Adeno TCR control	0,00	3,12	9,95
CD8 T +Adeno TCR w/ Adeno pept	227,26	1013,54	26,38
g/d +Adeno TCR+CD8a control	5,54	4,09	3,34
g/d +Adeno TCR+CD8a Adeno pept	623,42	4959,13	120,11

IFNgamma (pg/ml)	Experiment 1	Experiment 2	Experiment 3
CD8 T +mock control	30,85	31,65	47,26
CD8 T +mock w/ Adeno pept	41,44	62,76	54,41
CD8 T +Adeno TCR control	28,33	51,20	238,30
CD8 T +Adeno TCR w/ Adeno pept	10967,19	15859,80	2945,61
g/d +Adeno TCR+CD8a control	220,03	21,17	43,69
g/d +Adeno TCR+CD8a Adeno pept	16911,49	19691,21	5590,96

Table S5B: raw data to Fig. 5B

IL-2 (pg/ml)	Experiment 1	Experiment 2	Experiment 3
CD8 T +mock control	9,73	8,20	4,67
CD8 T +mock w/ Adeno pept	13,77	8,96	4,94
CD8 T +Adeno TCR control	5,84	5,78	5,55
CD8 T +Adeno TCR w/ Adeno pept	283,20	425,75	198,02
g/d +Adeno TCR+CD8a control	0,39	0,42	1,46
g/d +Adeno TCR+CD8a Adeno pept	5,94	730,83	113,49

TNF (pg/ml)	Experiment 1	Experiment 2	Experiment 3
CD8 T +mock control	159,52	172,41	14,23
CD8 T +mock w/ Adeno pept	171,04	176,75	15,99
CD8 T +Adeno TCR control	108,28	162,44	45,39
CD8 T +Adeno TCR w/ Adeno pept	1868,57	3214,14	144,29
g/d +Adeno TCR+CD8a control	220,78	146,95	61,25
g/d +Adeno TCR+CD8a Adeno pept	2759,23	7050,26	467,43

IFNgamma (pg/ml)	Experiment 1	Experiment 2	Experiment 3
CD8 T +mock control	7921,90	3884,45	501,22
CD8 T +mock w/ Adeno pept	7402,41	4553,82	357,97
CD8 T +Adeno TCR control	4336,73	2810,99	825,17
CD8 T +Adeno TCR w/ Adeno pept	24582,78	25803,59	9113,38
g/d +Adeno TCR+CD8a control	3212,10	515,34	1063,76
g/d +Adeno TCR+CD8a Adeno pept	23927,20	24335,88	8401,40

Table S6: raw data to Fig. 6

				Donor 1	Donor 2	Donor 3	Donor 4
CD8 T	M1/A1 TCR	w/o pept	Il-2	1,28	0,46	0,47	0,18
			TNF	0,67	0,24	0,44	0,49
			IFNgamma	1,83	0,58	0,78	1,8
		w/ pept	Il-2	1,92	0,66	0,52	0,13
			TNF	0,71	0,48	0,86	0,51
			IFNgamma	2,04	1,35	1,23	1,45
CD8 T	HAdV/A1 TCR	w/o pept	Il-2	1,07	1,05	0,87	0,09
			TNF	0,71	0,58	0.93	0,27
			IFNgamma	2,58	2,24	1,52	0,97
		w/ pept	Il-2	5,37	4,64	2,71	1,97
			TNF	22,81	12,78	11,91	7,67
			IFNgamma	47,96	39,99	29,71	20,48
g/d T	M1/A1 TCR	w/o pept	Il-2		0,44	0,93	0,34
			TNF		3,19	2,1	2,39
			IFNgamma		7,25	2,62	10,32
		w/ pept	Il-2		0,39	0,54	0,28
			TNF		3,32	2,26	4,13
			IFNgamma		7,44	2,9	16,24
g/d T	HAdV/A1 TCR	w/o pept	Il-2		0,36	0,28	0,17
			TNF		3,7	1,92	1,57
			IFNgamma		8,14	2,48	6,62
		w/ pept	Il-2		2,07	2,01	1,21
			TNF		28,14	25,96	9,5
			IFNgamma		50,08	33,05	26,52

Table S7A : raw data to Fig. 7A

Autologous system

		Exp. 1	Exp. 2	Exp.3	Exp. 4	Exp. 5	Exp. 6
CD8 M1/A1 TCR mDC empty	1:60	-7,863	3,988	1,28	1,047	6,386	7,601
	1:20	-4,983	-0,443	1,38	1,222	3,08	8,076
	1:6	-7,863	0,148	0,492	1,396	5,034	8,789
	1:2	-4,097	6,352	1,28	-0,524	1,127	3,325
CD8 HAdV/A1 TCR mDC empty	1:60	-6,977	2,511	2,86	1,919	3,681	9,739
	1:20	-11,406	-0,443	0,295	1,571	3,381	7,601
	1:6	-4,7619	1,625	0,394	-1,745	1,578	5,463
	1:2	-7,198	1,329	1,28	-0,175	2,179	3,8
CD8 M1/A1 TCR mDC+Adeno pept.	1:60	1,825	0,875	-0,139	-5,81	2,013	9,71
	1:20	-2,255	1,313	-4,596	3,267	4,866	10,68
	1:6	-2,47	1,969	0,696	6,897	1,342	13,59
	1:2	2,256	2,188	-6,267	11,978	0,671	4,531
CD8 HAdV/A1 TCR mDC+Adeno pept.	1:60	48,66	32,604	33,008	38,476	49,66	53,074
	1:20	45,65	30,853	25,766	46,824	41,275	49,51
	1:6	37,91	15,755	18,245	27,586	27,685	29,45
	1:2	10,2	7,44	5,432	15,608	13,758	16,83
CD8 M1/A1 TCR mDC+adenovirus	1:60	6,94	-3,741	10,311	3,321	4,88	9,615
	1:20	5,01	1,701	8,551	6,088	5,919	11,859
	1:6	2,47	0,34	2,782	6,088	6,957	15,385
	1:2	0,69	1,701	1,637	3,506	2,596	8,974
CD8 HAdV/A1 TCR mDC+adenovirus	1:60	35,16	24,83	25,2	19,926	25,65	46,47
	1:20	29,76	16,67	21,11	17,712	24,195	28,53
	1:6	14,88	12,585	10,966	9,963	14,226	28,21
	1:2	6,09	13,946	7,201	4,613	11,111	21,795

Table S7B: raw data to Fig. 7B

Autologous system

		Exp. 1	Exp. 2	Exp. 3	Exp. 4	Exp. 5	Exp. 6
g/d M1/A1 TCR mDC empty	1:60	-10,963	3,98	0,98	-0,17	3,83	8,79
	1:20	-4,54	0,443	-1,77	-1,396	2,93	-0,71
	1:6	-6,98	2,81	0,79	1,22	0,53	0,95
	1:2	-3,211	4,579	-0,89	-2,44	-0,83	-1,66
g/d HAdV/A1 TCR mDC empty	1:60	-5,65	0,148	-0,1	-1,92	4,88	5,46
	1:20	-10,08	-0,738	0,689	-1,396	2,48	2,61
	1:6	-5,87	0,148	0,1	-1,396	0,83	-0,95
	1:2	-6,53	-0,148	0,1	0,17	0,225	-1,19
g/d M1/A1 TCR mDC+Adeno pept.	1:60	-3,11	2,63	-10,17	-1,09	3,86	1,618
	1:20	-3,97	1,97	-0,42	1,81	2,01	-2,96
	1:6	-3,323	0,66	-11	1,09	0,839	1,29
	1:2	-3,33	2,63	-5,99	4,36	0,168	4,21
g/d HAdV/A1 TCR mDC+Adeno pept.	1:60	59,61	43,107	48,33	30,85	49,33	62,78
	1:20	57,25	32,17	34,96	56,99	39,77	66,02
	1:6	52,74	19,69	26,6	38,48	26,01	56,31
	1:2	31,04	14,44	15,18	18,15	13,26	25,89
g/d M1/A1 TCR mDC+adenovirus	1:60	3,55	5,78	2,455	2,77	6,33	-3,53
	1:20	2,62	3,06	3,76	1,11	4,04	0,96
	1:6	-0,31	-1,7	2,78	1,85	1,77	1,92
	1:2	0,54	1,02	0,98	-0,55	1,14	1,28
g/d HAdV/A1 TCR mDC+adenovirus	1:60	29,22	24,83	24,06	20,48	24,61	41,35
	1:20	26,75	25,51	17,51	16,61	17,34	36,86
	1:6	15,57	22,19	12,77	10,7	15,05	29,81
	1:2	9,33	4,42	10,47	7,75	13,4	15,71

Table S7C: raw data to Fig. 7C

Allogeneic system

CD8 M1/A1 TCR mDC empty

	Exp. 1	Exp. 2	Exp. 3	Exp. 4
1:60	4,363	2,74	17,11	3,46
1:20	1,745	5,18	13,46	0,07
1:6	0,873	1,57	9,26	2,28
1:2	-0,698	1,46	3,93	0,96

CD8 HAdV/A1 TCR mDC empty

1:60	2,967	7,63	21,879	1,69
1:20	1,396	2,85	13,18	0,96
1:6	-0,175	1,1	11,22	0,96
1:2	1,047	-0,99	6,17	1,98

CD8 M1/A1 TCR mDC+Adeno pept.

1:60	8,59	4,46	14,01	2,17
1:20	5,02	6,44	13,45	0,5
1:6	6,32	7,68	9,24	5,17
1:2	-1,78	0,74	4,48	2,67

CD8 HAdV/A1 TCR mDC+Adeno pept.

1:60	47,49	37,67	31,37	45,37
1:20	46,52	40,4	25,49	43,87
1:6	38,41	19,08	24,65	29,69
1:2	9,89	9,17	8,4	13,34

CD8 M1/A1 TCR mDC+adenovirus

1:60	6,76	7,38	13,08	9,87
1:20	2,02	6,24	12,05	16,45
1:6	2,54	6,52	10,77	17,11
1:2	-1,01	3,26	6,67	19,08

CD8 HAdV/A1 TCR mDC+adenovirus

1:60	25,53	25,67	32,82	30,92
1:20	16,74	18,44	28,72	26,97
1:6	13,69	16,03	19,74	25,66
1:2	6,26	12,34	9,23	10,53

Table S7D: raw data to Fig. 7D
Allogeneic system

Group	Ratio	Exp. 1	Exp. 2	Exp. 3	Exp. 4
g/d M1/A1 TCR mDC empty	1:60	-0,52	2,5	9,54	0,52
	1:20	-0,17	1,456	1,4	-0,96
	1:6	-0,17	1,57	1,4	-1,25
	1:2	-0,52	0,757	0,28	-0,66
g/d HAdV/A1 TCR mDC empty	1:60	-0,7	2,15	14,02	0,515
	1:20	1,05	1,22	5,05	-0,37
	1:6	-1,57	1,57	0,56	0,07
	1:2	-1,22	0,52	0,84	1,1
g/d M1/A1 TCR mDC+Adeno pept.	1:60	1,78	1,49	2,8	-0,17
	1:20	3,73	1,73	7	-1,33
	1:6	7,29	-2,23	3,08	-2,67
	1:2	1,13	-7,19	0	0,83
g/d HAdV/A1 TCR mDC+Adeno pept.	1:60	61,1	38,17	38,38	56,71
	1:20	66,29	45,85	30,53	45,54
	1:6	55,59	24,78	25,49	45,54
	1:2	21,88	16,6	7,28	36,2
g/d M1/A1 TCR mDC+adenovirus	1:60	-2,03	4,96	5,13	12,5
	1:20	-2,2	3,69	5,9	8,55
	1:6	-1,69	2,411	-1,28	5,92
	1:2	-0,85	2,12	0	0,66
g/d HAdV/A1 TCR mDC+adenovirus	1:60	19,95	22,13	30,26	50,66
	1:20	19,44	18,01	20,77	45,39
	1:6	14,71	12,199	19,49	38,82
	1:2	7,27	8,79	7,69	-1,31
